# Role of the Heme Activator Protein Complex in the Sexual Development of Cryptococcus neoformans

**DOI:** 10.1128/msphere.00170-22

**Published:** 2022-05-31

**Authors:** Jin-Young Kim, Yong-Sun Bahn

**Affiliations:** a Department of Biotechnology, Yonsei University, Seoul, Republic of Korea; University of Georgia

**Keywords:** Mating, Hap2/Hap3/Hap5/HapX, *C. neoformans*, CCAAT-binding HAP complex, Cpk1 MAPK pathway, *Cryptococcus neoformans*

## Abstract

The CCAAT-binding heme activator protein (HAP) complex, comprising the DNA-binding heterotrimeric complex Hap2/3/5 and transcriptional activation subunit HapX, is a key regulator of iron homeostasis, mitochondrial functions, and pathogenicity in Cryptococcus neoformans, which causes fatal meningoencephalitis. However, its role in the development of human fungal pathogens remains unclear. To elucidate the role of the HAP complex in C. neoformans development, we constructed *hap2*Δ, *hap3*Δ, *hap5*Δ, and *hapX*Δ mutants and their complemented congenic *MAT*α H99 and *MAT***a** YL99**a** strains. The HAP complex plays a conserved role in iron utilization and stress responses in cells of both mating types. Deletion of any of the HAP complex components markedly enhances filamentation during bisexual mating. However, the Hap2/3/5 complex, but not HapX, is crucial in repressing pheromone production and cell fusion and is thus a critical repressor of sexual differentiation of C. neoformans. Interestingly, deletion of the heterotrimeric complex transcriptionally regulated both positive and negative regulators in the pheromone-responsive Cpk1 mitogen-activated protein kinase (MAPK) pathway. Chromatin immunoprecipitation-quantitative PCR analysis revealed that the HAP complex physically bound to the CCAAT motif of the *CRG1* and *GPA2* promoter regions. Notably, the HAP complex was differentially localized depending on the mating type in basal conditions; it was enriched in the nuclei of *MAT*α cells but diffused in the cytoplasm of *MAT***a** cells. Interestingly, however, a portion of the HAP complex in both mating types relocalized to the cell membrane during mating. In conclusion, the Hap2/3/5 heterotrimeric complex and HapX play major and minor roles, respectively, in repressing the sexual development of C. neoformans in association with the Cpk1 MAPK pathway.

**IMPORTANCE** Cryptococcus neoformans isolates are of two mating types: *MAT*α strains, which are predominant, and *MAT***a** strains, isolated from the sub-Saharan African region, where cryptococcosis is most abundant and severe. Here, we demonstrated the function of the CCAAT-binding HAP complex (Hap2/3/5/X) as a transcriptional repressor of Cpk1 pathway-related genes in cells of both mating types. Deletion of any HAP complex component markedly enhanced filamentation without affecting normal sporulation. In particular, deletion of the DNA-binding HAP complex components (Hap2/3/5), but not HapX, markedly enhanced pheromone production and cell fusion efficiency, validating its repressive role in the early stage of mating in C. neoformans. The HAP complex regulates the expression of both negative and positive mating regulators and is thus crucial for the regulation of the Cpk1 MAPK pathway during mating. This study provides insights into the complex signaling networks governing the sexual differentiation of C. neoformans.

## INTRODUCTION

The CCAAT box is one of the most frequently occurring motifs in eukaryotic promoter regions (1). It is highly conserved and is present in approximately 30% of eukaryotic promoter regions, located approximately 60 to 100 bp upstream of the transcription start sites (2–4). Recognition of the CCAAT box by numerous DNA-binding proteins allows transcriptional activation, which affects gene expression (5, 6). DNA-binding proteins that recognize and bind to the CCAAT box form the CCAAT-binding complex (CBC) and are generally composed of a heterotrimeric complex that is conserved from yeasts to vertebrates (7–9). CBCs are known to regulate various cellular functions, such as primary/secondary metabolism, cell development, and stress responses (9–12). CCAAT motifs are among the regulatory sequences that have low nucleosome occupancy at the functional binding sites, ensuring that the genomic DNA is readily accessible to other transcription factors (13–15). These data demonstrate the role of the CBC in promoter recognition and organization.

In mammals, nuclear factor Y (NF-Y) was the first CBC to be identified and studied (7, 16). NF-Y plays critical and often indispensable roles in gene regulation, respiratory metabolism, cell proliferation, and early embryonic development by binding to the CCAAT box (17–21). NF-Y is composed of three evolutionarily conserved subunits: NF-YA, NF-YB, and NF-YC (22). Crystallographic structural analysis revealed that NF-YB interacts with NF-YC to form a tight heterodimer through a histone fold domain (HFD), which is responsible for creating a platform facilitating the binding and bending of DNA (14, 23). NF-YA consists of two α-helical domains; the N-terminal domain is required for interaction with the NF-YB/NF-YC heterodimer, and the C-terminal domain is involved in recognizing and binding to CCAAT elements. Furthermore, both NF-YA and NF-YC contain the activation domain for this heterotrimeric complex (9, 24). Many transcription factors belonging to the NF-Y family have been identified in eukaryotic species. In yeast species such as Cryptococcus neoformans, Saccharomyces cerevisiae, Candida albicans, and Candida glabrata, the NF-Y family has been designated the heme activator protein (HAP) complex (9).

The HAP complex in S. cerevisiae has been identified as a heme-dependent transcriptional activator (25). It is composed of four subunits (Hap2, Hap3, Hap4, and Hap5) and is thus structurally and functionally different in DNA binding and activation from heterotrimeric mammalian NF-Y (26–28). The DNA-binding domain of the HAP complex consists of three essential subunits, Hap2, Hap3, and Hap5 (abbreviated Hap2/3/5), and the activation domain is conferred by Hap4 (27–31). Although Hap2, -3, and -5 are expressed constitutively and present as a heterotrimeric complex, Hap4 must be recruited to trigger transcriptional activation (27). Once Hap2/3/5 binds to the CCAAT motif, the recruitment domain present in Hap5 recruits Hap4 to associate with the heterotrimer. Other domains conferring this Hap4 recruitment within the HAP complex are yet to be discovered (9, 29, 30). Hap4 homologs have only been found in fungi and not in higher organisms, suggesting that these three subunits are sufficient for DNA binding and activation in humans (9, 27, 31).

The HAP complex has been functionally characterized in the basidiomycete C. neoformans, an etiological agent of fungal meningoencephalitis responsible for more than 181,000 deaths annually worldwide (32–34). HapX was previously discovered to play an important role in iron homeostasis as a regulatory subunit of the CBC in C. neoformans. It negatively regulates the expression of genes involved in respiratory and tricarboxylic acid (TCA) cycle functions under low-iron conditions and positively regulates genes involved in iron uptake. Furthermore, deletion of *HAP3*, *HAP5*, or *HAPX* attenuates the growth of C. neoformans on hemin, proving that they contribute to iron utilization from hemin (34). Thus far, most studies on the HAP complex in fungi have focused on its role in iron utilization. However, the role of the HAP complex in fungal development and differentiation remains unclear.

Cryptococcus neoformans has a bipolar mating type system that consists of α and **a** mating types and can undergo bisexual and unisexual differentiation. In the bisexual mating system, cells of opposite mating types recognize mating pheromones, fuse together with the maintenance of two nuclei (plasmogamy), and form hyphae with fused clamp connections to ensure proper segregation of nuclei. Formed at the tips of the hyphae is the basidium, where two nuclei of opposite mating types undergo nuclear fusion (karyogamy). Then diploid cells undergo meiosis to produce four haploid nuclei and form four chains of basidiospores at the tip of the basidium (35, 36). Despite the well-defined sexual mating process, the majority of naturally isolated C. neoformans strains have the α mating type, suggesting that bisexual mating may not be predominant in nature (37). Nevertheless, bisexual mating appears to be important for understanding the pathogenesis of cryptococcosis, because most of *MAT***a** C. neoformans strains have been isolated in the sub-Saharan African region, where cases of cryptococcosis are most abundant and severe (32, 38).

The Cpk1 mitogen-activated protein kinase (MAPK) pathway is known to modulate unisexual/bisexual mating and dimorphic switching in C. neoformans. Disruption of the Cpk1 MAPK pathway components completely abolishes all the aforementioned processes during mating, confirming its absolute requirement for the pathway in the sexual development of C. neoformans (39–42). Nevertheless, our systematic functional analysis of transcription factors and kinases in C. neoformans revealed that a number of other signaling components are also involved in mating processes (33, 43), suggesting that signaling networks regulating mating are much more complex than originally expected. We previously found Hap2 to be negatively involved in the mating process of C. neoformans by repressing pheromone production (33). However, it remains unclear how the other HAP complex components play a similar role in mating and, if so, how they regulate the mating process in relation to the Cpk1 MAPK pathway. In this study, we demonstrated that the Hap2/3/5 heterotrimeric complex and HapX play major and minor roles, respectively, in repressing the sexual development of C. neoformans by regulating the pheromone-responsive Cpk1 MAPK pathway.

## RESULTS

### Construction of HAP complex mutants in C. neoformans serotype A *MAT*α and *MAT*a strains.

**FIG 1:**
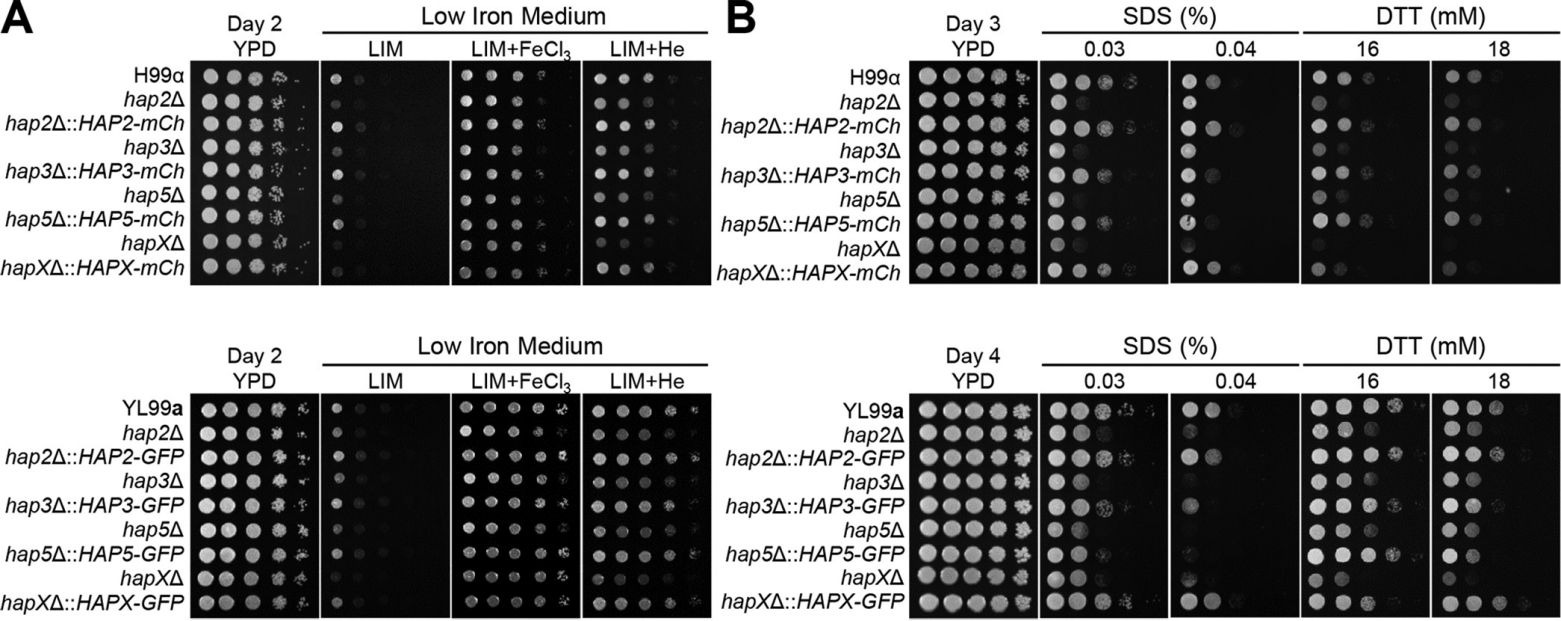
Phenotypic traits of the HAP mutants in both *MAT*α and *MAT***a** strains on various stress media. Cryptococcus neoformans HAP complex mutants of *MAT*α and *MAT***a** strains were cultured in liquid YPD medium at 30°C overnight. The cells were serially diluted 10-fold and spotted onto YPD medium, low-iron medium (LIM), or LIM supplemented with 100 μM FeCl_3_ or 10 μM hemin (A). The plates were incubated at 30°C for 2 days. (B) YPD medium containing the cell wall/membrane stressor, sodium dodecyl sulfate (SDS), and the ER stressor dithiothreitol (DTT).

To elucidate the role of the HAP complex in the sexual differentiation of C. neoformans, genes encoding Hap2, Hap3, Hap5, and HapX were deleted in the *MAT*α H99 strain and its congenic *MAT***a** YL99**a** strain using nourseothricin acetyltransferase (*NAT*) and neomycin phosphotransferase (*NEO*) markers, respectively (see Fig. S1 in the supplemental material). To validate mutant phenotypes and perform localization and chromatin immunoprecipitation (ChIP) analyses, we constructed the following complemented strains for all *MAT*α and *MAT***a** HAP mutants with *mCherry-* or *GFP-*tagged wild-type alleles (*GFP* encodes green fluorescent protein) (Fig. S2). All fluorescent protein-tagged HAP components appeared to be functional because each tagged allele restored the wild-type phenotype of each mutant, as shown later. First, we examined whether the HAP complex in *MAT*α and *MAT***a** cells has differential roles in iron utilization and stress responses (Fig. 1). Previous studies have shown that the growth of *MAT*α *hap3*Δ, *hap5*Δ, and *hapX*Δ mutants on a low-iron medium is retarded but becomes normal upon the addition of the feroxamine or ferric chloride while showing partial restoration of growth on a medium supplemented with hemin as the sole iron source (34). We found that all the HAP mutants constructed in the *MAT***a** background strain also displayed similar growth defects on low-iron medium, restoration of near-normal growth in the presence of the ferric chloride, and partial restoration of growth in the presence of hemin (Fig. 1). These defects were restored in the complemented strains (Fig. 1A). We previously reported that the *hap2*Δ mutant exhibit increased or decreased susceptibility to diverse stress agents (33). In this study, we found that all the HAP complex mutants in *MAT*α and *MAT***a** strains exhibit increased susceptibility to a cell wall/membrane stressor such as sodium dodecyl sulfate (SDS) and an endoplasmic reticulum (ER) stressor like dithiothreitol (DTT) (Fig. 1B). These results show that the HAP complex plays a conserved role in iron utilization and stress responses in both mating types of C. neoformans.

10.1128/msphere.00170-22.1 FIG S1 Disruption of Cryptococcus neoformans HAP complex in serotype A *MAT*α H99 and *MAT***a** YL99**a**. Shown is disruption of the *HAP3*, *HAP5*, and *HAPX* genes in the *MAT*α H99 strain (A to C) and *HAP2*, *HAP3*, *HAP5*, and *HAPX* genes in the *MAT***a** YL99**a** strain (D to G). The correct gene disruption was verified by Southern blotting using genomic DNAs digested with the indicated restriction enzymes. The representative strains used in this study are shown in bold. Download FIG S1, PDF file, 0.6 MB. Copyright © 2022 Kim and Bahn. 2022 Kim and Bahn. https://creativecommons.org/licenses/by/4.0/ This content is distributed under the terms of the Creative Commons Attribution 4.0 International license.

10.1128/msphere.00170-22.2 FIG S2 Construction of HAP complex-complemented strains in serotype A *MAT*α H99 and *MAT***a** YL99**a**. The complemented strains were confirmed via targeted diagnostic PCR, using the specific primer pairs listed in Table S2. Download FIG S2, PDF file, 0.4 MB. Copyright © 2022 Kim and Bahn. 2022 Kim and Bahn. https://creativecommons.org/licenses/by/4.0/ This content is distributed under the terms of the Creative Commons Attribution 4.0 International license.

### Role of HAP complex as a mating repressor.

**FIG 2:**
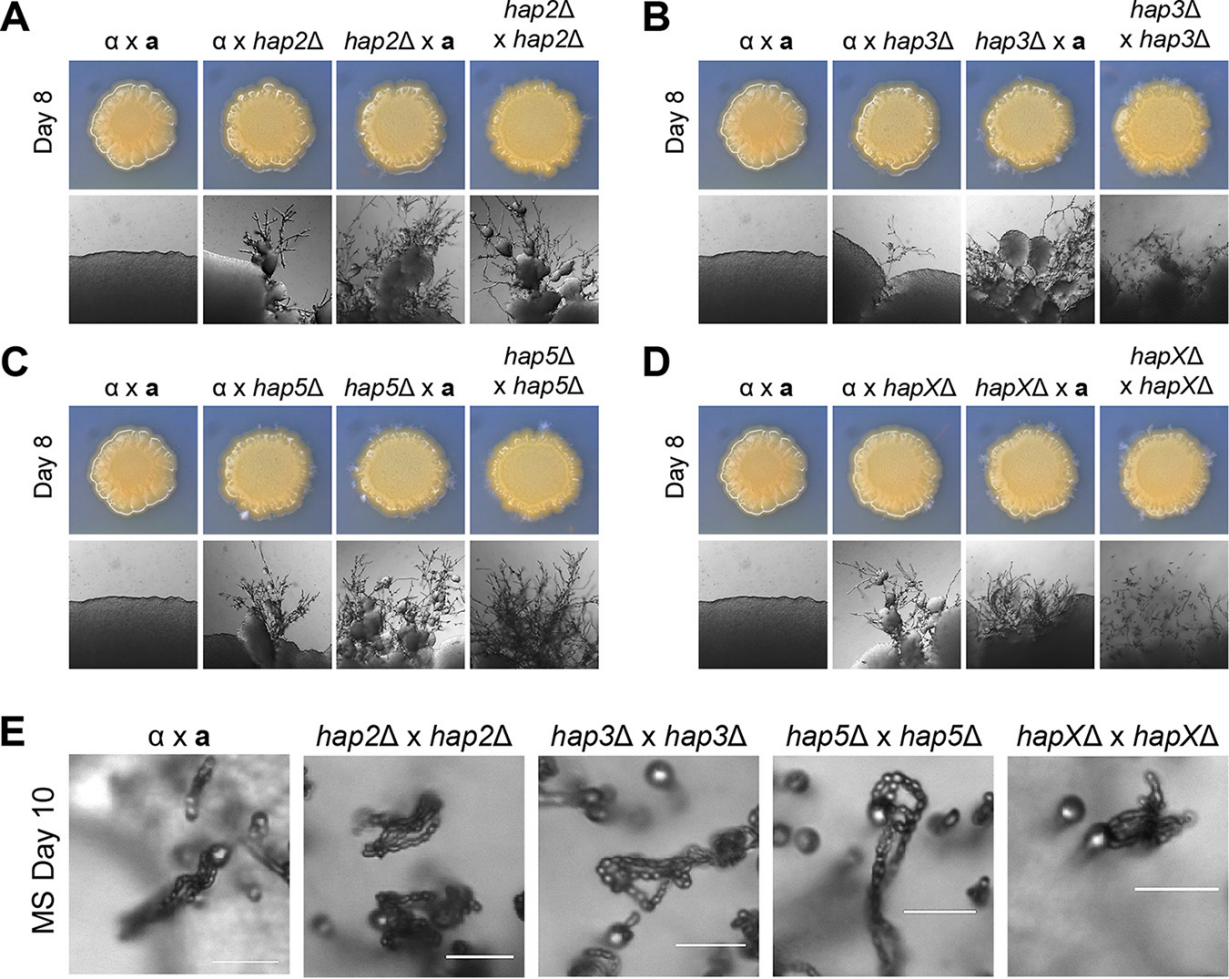
Filamentation and basidiospore formation of the HAP complex mutants. HAP complex mutants of the *MAT*α and *MAT***a** strains were cultured in liquid YPD medium at 30°C overnight. The cells were diluted to 10^7^ cells/ml, and the indicated *MAT*α and *MAT***a** strains were cocultured in V8 medium (pH 5.0) for 7 to 10 days at room temperature in the dark. The strains used for the mating assay are as follows: for panel A, α (H99) × **a** (YL99**a**), α (H99) × **a** *hap2*Δ (YSB5081), α *hap2*Δ (YSB1104) × **a** (YL99**a**), and α *hap2*Δ (YSB1104)  × **a** *hap2*Δ (YSB5081); for panel B, α (H99) × **a** (YL99**a**), α (H99) × **a** *hap3*Δ (YSB7423), α *hap3*Δ (YSB7417) × **a** (YL99**a**), and α *hap3*Δ (YSB7417)  × **a** *hap3*Δ (YSB7423); for panel C, α (H99) × **a** (YL99**a**), α (H99) × **a** *hap5*Δ (YSB7425), α *hap5*Δ (YSB7420) × **a** (YL99**a**), and α *hap5*Δ (YSB7420)  × **a** *hap5*Δ (YSB7425); and for panel D, α (H99) × **a** (YL99**a**), α (H99) × **a** *hapX*Δ (YSB7432), α *hapX*Δ (YSB7992) × **a** (YL99**a**), and α *hapX*Δ (YSB7992)  × **a** *hapX*Δ (YSB7432). (E) The HAP complex mutants were cocultured on MS medium for 10 days at room temperature in the dark and visualized under a microscope for spore formation. Bars, 20 μm.

To elucidate the role of the HAP complex in the sexual reproduction of C. neoformans, each *MAT*α HAP mutant was coincubated with the *MAT***a** wild-type YL99**a** strain (unilateral mating) or HAP mutant (bilateral mating) in V8 medium (Fig. 2A to D). Both unilateral and bilateral mating with the *hap2*Δ, *hap3*Δ, *hap5*Δ, and *hapX*Δ mutants led to a marked increase in filamentation (Fig. 2A to D). The enhanced filamentation of the HAP mutants led us to question whether sporulation remained intact. Four chains of basidiospores were formed normally at the tips of the basidium in bilateral mating of all HAP mutants, signifying that the HAP complex does not affect the sporulation process in C. neoformans (Fig. 2E). Collectively, these results indicate that all HAP components play conserved roles in repressing mating in C. neoformans.

### HAP complex represses pheromone production.

**FIG 3:**
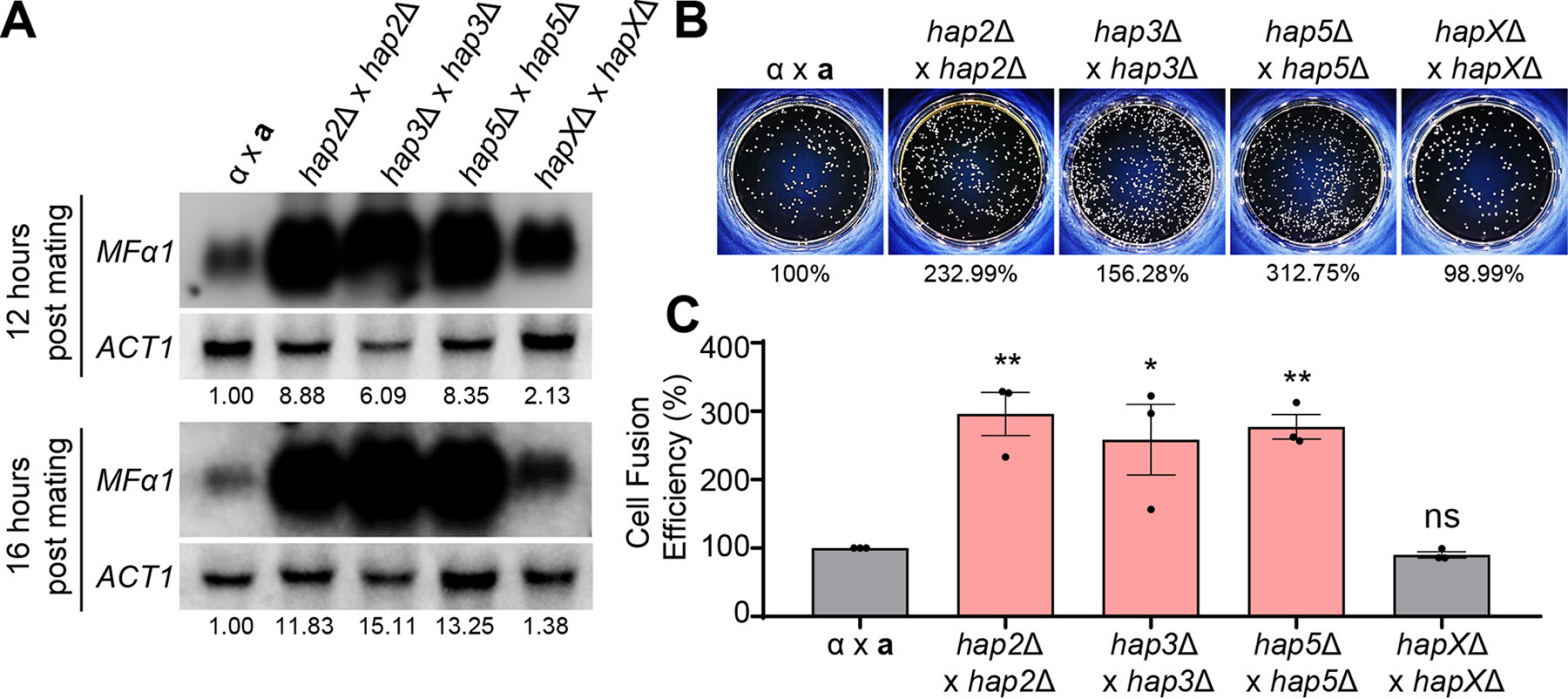
Pheromone production levels and cell fusion efficiencies of the HAP complex mutants. (A) Northern blot analysis was employed to monitor the expression levels of the *MFα1* pheromone gene using RNA isolated from cocultures of the HAP mutants in V8 medium. The RNA was isolated from cocultures at 12 and 16 h postmating. The expression levels of the *MFα1* gene were normalized by the expression levels of the housekeeping gene *ACT1*, and the expression levels were further analyzed by setting the pheromone expression levels of α × **a** as 1. (B) Representative data are shown for the cell fusion assay of the HAP complex. The percentages take into account the mean of one biological replicate. (C) Cell fusion assay was performed by using YSB119 for *MAT*α and YSB121 for *MAT***a** controls. The data plots are for three biological replicates, and the error bars indicate the standard errors of the means (SEM). The statistical significance of difference was determined using one-way analysis of variance (ANOVA) with Bonferroni’s multiple-comparison test: *, *P* < 0.05; **, *P < *0.01. ns, not significant.

The finding that the deletion of any HAP complex component markedly enhanced filamentation of C. neoformans suggests that the HAP complex may play a repressive role in promoting pheromone production and cell fusion (or both), which represents the early stage of mating. To address this issue, we measured the expression level of the *MFα1* pheromone gene during bilateral mating between *MAT*α and *MAT***a** wild-type organisms and HAP mutants. Bilateral mating with *hap2*Δ, *hap3*Δ, and *hap5*Δ mutants led to a drastic increase (6- to 15-fold) in pheromone levels 12 to 16 h postmating, whereas mating with *hapX*Δ mutants yielded only a modest increase (~2-fold) in pheromone production (Fig. 3A). This result strongly suggests that the Hap2/3/5 heterotrimeric complex and HapX play major and minor roles, respectively, in repressing pheromone production during mating.

Once pheromones are released and recognized by cells of the opposing mating type, these cells undergo plasmogamy, also known as cell fusion, in which the protoplasm of two parent cells fuses together without the nuclei fusing, creating a cell with two haploid nuclei in proximity (dikaryon) (44). To address whether increased pheromone production in the HAP mutants resulted in enhanced cell fusion, we measured the cell fusion efficiency by monitoring the *NAT NEO* double-marked dikaryotic CFU upon mating between *NAT-*marked *MAT*α and *NEO-*marked *MAT***a** control strains or HAP mutants (Fig. 3B and C). Bilateral mating with *hap2*Δ, *hap3*Δ, and *hap5*Δ mutants led to a significant increase in cell fusion efficiency (1.5- to 3-fold) (Fig. 3C), which reflected an increased pheromone production in the corresponding mutants (Fig. 3A). In contrast, cell fusion efficiency did not increase during bilateral mating between *hapX*Δ mutants (Fig. 3C), in agreement with only a modest increase in pheromone production during bilateral mating with *hapX*Δ mutants. Thus, the drastic increase in pheromone production after the deletion of Hap2/3/5 seemed to be the cause of increased cell fusion, leading to robust filamentation. These data represent the direct correlation between mating pheromone production, cell fusion efficiency, and filamentation, reinforcing the fact that the Hap complex acts as a repressor in the early stage of sexual reproduction in C. neoformans.

### Role of the HAP complex as a positive and negative regulator of the Cpk1 MAPK pathway.

**FIG 4:**
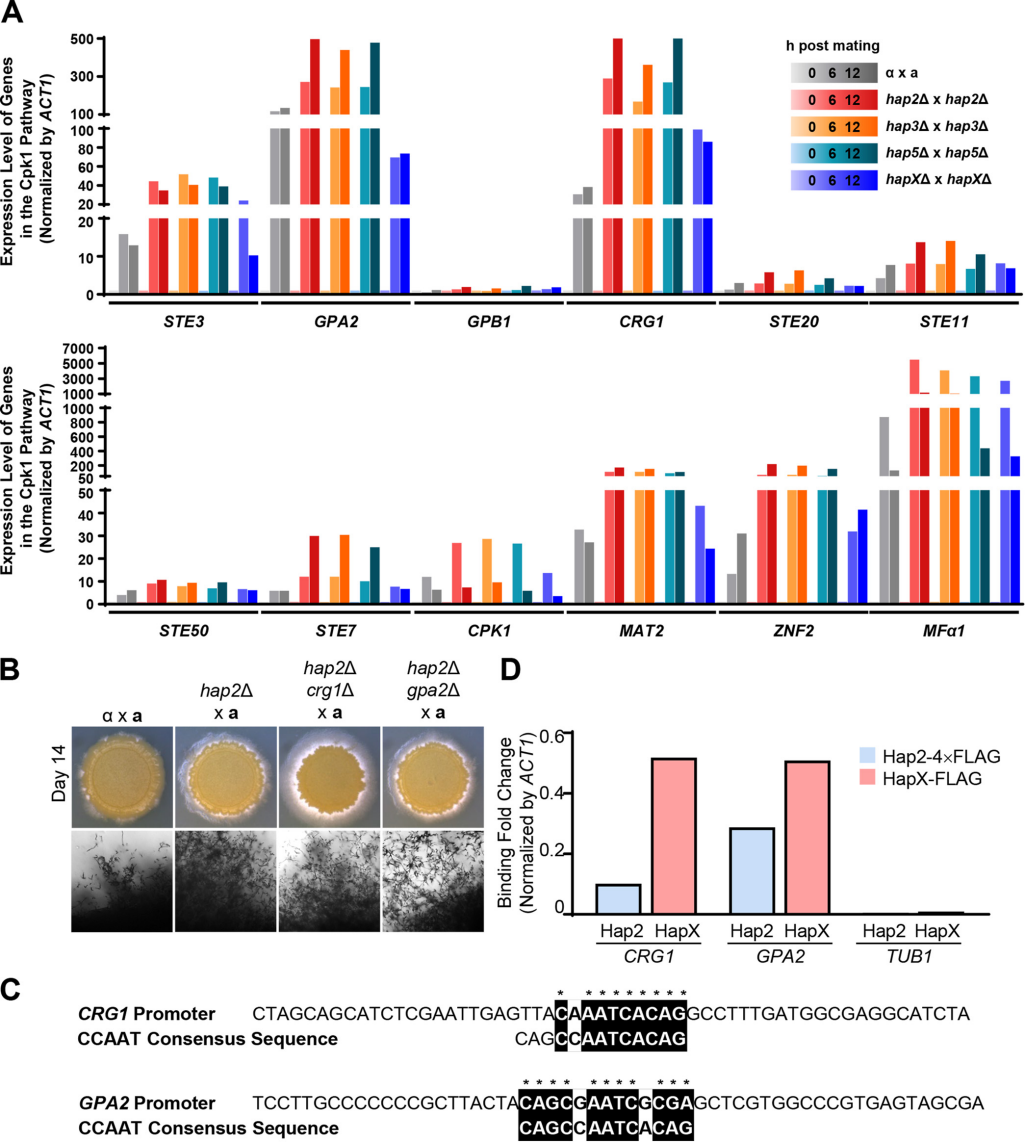
Expression levels of genes in the Cpk1 pathway during bilateral mating of HAP complex mutant strains and HAP complex’s ability to bind to their promoters. (A) qRT-PCR was employed to monitor the expression levels of the various genes in the Cpk1 pathway using RNA isolated from cocultures of the HAP mutants on V8 medium. The RNA was isolated from cocultures at 0, 6, and 12 h following mating, and the expression levels of these gene were normalized by the expression levels of the housekeeping gene *ACT1*. The expression level of these genes at 0 h was set as 1. (B) Cryptococcus neoformans cells were cultured in liquid YPD medium at 30°C overnight. The cells were spotted onto V8 medium (pH 5.0) and incubated in the dark for 14 days. Representative edges of the mating patches were photographed at a magnification of ×100. The strains used for the mating assay were as follows: α (H99) × **a** (YL99**a**), α *hap2*Δ (YSB1104) × **a** (YL99**a**), α *hap2*Δ *crg1*Δ (YSB8884) × **a** (YL99**a**), and α *hap2*Δ *gpa2*Δ (YSB8890) × **a** (YL99**a**). (C) The CCAAT consensus sequence bound by the HAP complex in C. neoformans was found in the promoters of *CRG1* and *GPA2* using ClustalX. (D) ChIP-qPCR was performed under basal conditions for the *MAT*α *hap2*Δ::*HAP2*-*4×FLAG* (YSB8907) and *MAT*α *hapX*Δ::*HAPX-FLAG* strains (6). The binding fold change of Hap2 and HapX to *CRG1*, *GPA2*, and *TUB1* was calculated as follows: 2^−[IP(gene − actin) − WCE(gene − actin)]^.

Pheromone production, cell fusion, and filamentation are all governed by the Cpk1 MAPK pathway in C. neoformans (36, 39–42, 44). Therefore, we hypothesized that the HAP complex may act as a repressor in controlling the expression of Cpk1 MAPK signaling components. To test this possibility, we monitored the expression levels of Cpk1 MAPK signaling components, including *STE3* (a pheromone receptor), *GPB1* (G-protein β subunit), *GPA2* (G-protein α subunit), *CRG1* (a regulator of G-protein signaling), *STE20* (a p21-activated kinase), *STE50* (a scaffold/adaptor protein), *STE11* (MAPK kinase kinase [MAPKKK]), *STE7* (MAPKK), *CPK1* (MAPK), *MAT2* (Cpk1-dependent transcription factor), and *ZNF2* (master regulator of hyphal growth) during bilateral mating with *MAT*α and *MAT***a** wild-type strains against bilateral mating of the HAP complex genes, to discover genes that were differentially regulated by the HAP complex during mating. Quantitative reverse transcription-PCR (qRT-PCR) revealed that the expression of *GPA2*, *CRG1*, *MAT2*, *ZNF2*, and *MFα1* was greatly induced during mating, whereas kinase genes such as *STE7* and *CPK1* were only modestly induced (Fig. 4A). We found that the mating-induced levels of *GPA2*, *CRG1*, *MAT2*, *ZNF2*, and *MFα1* were greater in the bilateral mating of the *hap2*Δ, *hap3*Δ, and *hap5*Δ mutants than in the wild-type and *hapX*Δ mutant strains (Fig. 4A). The degree of induction of each mating gene was not statistically significant because of the batch-to-batch variation that naturally occurred during the mating experiment. However, the general trends of increase/decrease in the expression levels of these genes were similar for all three biological replicates (Fig. S3).

10.1128/msphere.00170-22.3 FIG S3 Expression levels of genes in the Cpk1 pathway during bilateral mating of HAP complex mutant strains. qRT-PCR was used to monitor the expression levels of various genes in the Cpk1 pathway using RNA isolated from cocultures of HAP mutants on V8 medium. RNA was isolated from cocultures at 0, 6, and 12 h following mating, and the expression levels of these genes were normalized to the expression levels of the housekeeping gene *ACT1*. The expression levels of these genes at 0 h were set at 1. Results for the second biological replicate (A) and third biological replicate (B) of this experiment are shown. Download FIG S3, PDF file, 0.06 MB. Copyright © 2022 Kim and Bahn. 2022 Kim and Bahn. https://creativecommons.org/licenses/by/4.0/ This content is distributed under the terms of the Creative Commons Attribution 4.0 International license.

The finding that the HAP complex affects the expression of both negative mating regulators (e.g., *CRG1*) and positive mating regulators (e.g., *GPA2*, *MAT2*, and *ZNF2*) proposes the involvement of the HAP complex in balanced regulation of the Cpk1 MAPK pathway during mating. To test this possibility, we constructed *hap2*Δ *crg1*Δ and *hap2*Δ *gpa2*Δ mutants (Fig. S4) and examined unilateral mating efficiency. Previous studies reported that *CRG1* acts as a repressor of filamentation (45) and *GPA2* acts as an activator of filamentation (46). The *hap2*Δ *crg1*Δ mutant showed even more enhanced filamentous growth than the *hap2*Δ mutant (Fig. 4B), indicating that the expression of *CRG1* was increased in the *hap2*Δ mutant for the negative feedback regulation of mating. However, the deletion of *GPA2* in the *hap2*Δ mutant led to filamentous growth similar to that of unilateral mating of *hap2*Δ (Fig. 4B), further supporting that the HAP complex repressed the Gpa2-dependent Cpk1 MAPK pathway.

10.1128/msphere.00170-22.4 FIG S4 Construction of *hap2*Δ *gpa2*Δ and *hap2*Δ *crg1*Δ mutant strains. (A) Schematic representation of the *GPA2* disruption strategy and confirmation of the deletion of the *GPA2* gene in the *MAT*α *hap2*Δ (YSB1104) mutant strain through Southern blot analysis. (B) Schematic representation of *CRG1* disruption strategy and confirmation of the deletion of the *CRG1* gene in the *hap2*Δ mutant strain through Southern blot analysis. Download FIG S4, PDF file, 0.2 MB. Copyright © 2022 Kim and Bahn. 2022 Kim and Bahn. https://creativecommons.org/licenses/by/4.0/ This content is distributed under the terms of the Creative Commons Attribution 4.0 International license.

As the Hap2/3/5 complex affected the expression levels of *CRG1* and *GPA2* during mating, we examined whether these genes contained a CCAAT-binding motif in their promoter sequences. Our investigation found a CCAAT-binding motif in the promoter regions of both genes (Fig. 4C). To validate whether the HAP complex binds directly to the CCAAT-binding motif of *CRG1* and *GPA2*, we performed ChIP-quantitative PCR (qPCR) analysis using the *MAT*α *hap2*Δ::*HAP2-4×FLAG-*complemented strain (Fig. S5) and the *MAT*α *hapX*::*NAT HAPX-FLAG-NEO* strain constructed by Do et al. (6). ChIP-qPCR analysis revealed that both Hap2-4×FLAG and HapX-FLAG specifically bound to the CCAAT-binding motifs of *CRG1* and *GPA2*, whereas they did not bind to the nonspecific promoter region of *TUB1* (Fig. 4D and Fig. S6).

10.1128/msphere.00170-22.5 FIG S5 Construction of FLAG-tagged Hap2 strain. (A) The targeted reintegration of the pHAP2-4**×**FLAG plasmid to *MAT*α *hap2*Δ (YSB1104) was confirmed using diagnostic PCR. (B) The functionality of the constructed strain was tested using immunoprecipitation of the Hap2-4**×**FLAG protein with an anti-FLAG antibody (IP: anti-FLAG). Download FIG S5, PDF file, 0.09 MB. Copyright © 2022 Kim and Bahn. 2022 Kim and Bahn. https://creativecommons.org/licenses/by/4.0/ This content is distributed under the terms of the Creative Commons Attribution 4.0 International license.

10.1128/msphere.00170-22.6 FIG S6 Binding of Hap2 and HapX to *CRG1* and *GPA2* promoters. Three biological replicates of ChIP-qPCR were used to examine the binding of the Hap2-4×FLAG and HapX-FLAG strains to the promoters of *CRG1*, *GPA2*, and *TUB1*. ChIP-qPCR was performed under basal conditions for *MAT*α *hap2*Δ::*HAP2*-*4****×****FLAG* (YSB8907) and *MAT*α *hapX*Δ::*HAPX-FLAG* strains (E. Do, Y. J. Cho, D. Kim, J. W. Kronstad, et al., Genetics 215:1171–1189, 2020, https://doi.org/10.1534/genetics.120.303270). The fold changes in binding of Hap2 and HapX to *CRG1, GPA2,* and *TUB1* were calculated as follows: 2^−[IP(gene − actin) – WCE (gene − actin)]^. Download FIG S6, PDF file, 0.05 MB. Copyright © 2022 Kim and Bahn. 2022 Kim and Bahn. https://creativecommons.org/licenses/by/4.0/ This content is distributed under the terms of the Creative Commons Attribution 4.0 International license.

Given the role of the HAP complex in the transcriptional regulation of genes in the Cpk1 MAPK pathway, we also examined whether the expression of the HAP complex itself is regulated during mating. To examine the expression levels of the HAP complex during mating, we performed qRT-PCR at 6 and 12 h following mating (Fig. S7). We found that the HAP complex transcript levels were constitutively expressed but did not vary during mating. Thus, the expression level of the HAP complex did not appear to directly impact the effects of mating.

10.1128/msphere.00170-22.7 FIG S7 Transcriptional regulation of the HAP complex during mating. The α (H99) and **a** (YL99**a**) strains were cultured in liquid YPD medium at 30°C overnight. The cells were coincubated for 0, 6, and 12 h on V8 medium and scraped at the corresponding time points. The expression of each HAP complex gene was examined using qRT-PCR by normalizing the gene expression levels with *ACT1*. Download FIG S7, PDF file, 0.04 MB. Copyright © 2022 Kim and Bahn. 2022 Kim and Bahn. https://creativecommons.org/licenses/by/4.0/ This content is distributed under the terms of the Creative Commons Attribution 4.0 International license.

### Changes in cellular localization of the HAP complex during mating.

**FIG 5:**
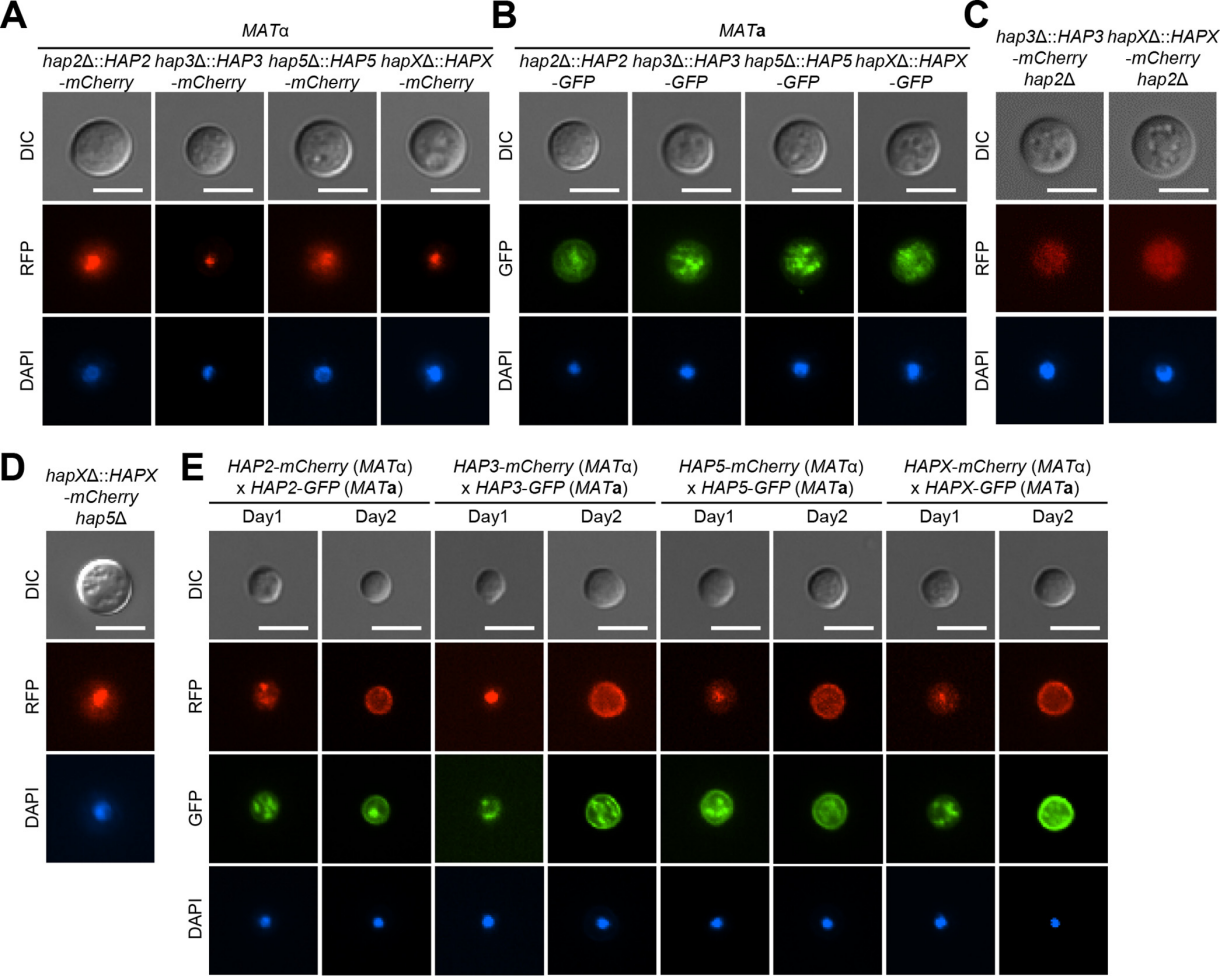
Cellular localization of the HAP complex. The strains were cultured in liquid YPD medium at 30°C overnight, fixed with formaldehyde, and stained with 4′,6-diamidino-2-phenylindole (DAPI) to visualize nuclei. Bars, 5 μm. (A) *MAT*α HAP complex-complemented strains with *mCherry* fluorescent tags were visualized under basal (non-mating) conditions. The strains used for localization visualization were *hap2*Δ::*HAP2*-*mCherry* (YSB6931), *hap3*Δ::*HAP3*-*mCherry* (YSB8541), *hap5*Δ::*HAP5*-*mCherry* (YSB8543), and *hapX*Δ::*HAPX*-*mCherry* (YSB8544). (B) *MAT***a** HAP complex-complemented strains with *GFP* fluorescent tags were visualized under basal conditions. The strains used for localization visualization were *hap2*Δ::*HAP2*-*GFP* (YSB8020), *hap3*Δ::*HAP3*-*GFP* (YSB9888), *hap5*Δ::*HAP5*-*GFP* (YSB9889), and *hapX*Δ::*HAPX*-*GFP* (YSB9658). (C) Localization of *MAT*α Hap3 and *MAT*α HapX in the absence of NLS-containing *HAP2* was visualized under basal conditions. The strains used were *MAT*α *hap3*Δ::*HAP3-mCherry hap2*Δ (YSB9660) and *MAT*α *hapX*Δ::*HAPX-mCherry hap2*Δ (YSB9890) (D) Localization of *MAT*α HapX in the absence of *HAP5* was visualized under basal conditions. The *MAT*α *hapX*Δ::*HAPX-mCherry hap5*Δ strain (YSB9891) was used. (E) For the indicated time periods, HAP complex mutants were cocultured in V8 medium in the dark. Cellular localization of the *MAT*α and *MAT***a** HAP complex subunits was visualized by fluorescence microscopy.

As the HAP complex appears to play the role of a transcriptional repressor and activator, we addressed whether it is constitutively localized to the nucleus or undergoes translocation between the cytoplasm and nucleus during mating. To address this question, we monitored the cellular localization of each HAP component by establishing bilateral mating between *MAT*α *hap2*Δ::*HAP2-mCherry*, *hap3*Δ::*HAP3-mCherry*, *hap5*Δ::*HAP5-mCherry*, and *hapX*Δ::*HAPX-mCherry* strains and the corresponding *MAT***a** *hap2*Δ::*HAP2-GFP*, *hap3*Δ::*HAP3-GFP*, *hap5*Δ::*HAP5-GFP*, and *hapX*Δ::*HAPX-GFP* strains. In S. cerevisiae, the Hap2, Hap3, and Hap5 subunits are assembled to form a heterotrimeric complex and then translocate to the nucleus via the nuclear localization signal (NLS) of Hap2, and without Hap2, the rest of the HAP complex is unable to localize to the nucleus (29, 47, 48). HapX is recruited to the nucleus by Hap5 and other domains in the HAP complex (9, 28–30). In this study, we found that all HAP complex components in the C. neoformans *MAT*α strain background were localized to the nucleus under nonmating basal conditions (Fig. 5A). Unexpectedly, however, the HAP complex in the *MAT***a** strain background was not particularly enriched in the nucleus but appeared to be diffused in organelles in the cytoplasm (Fig. 5B). As shown in the S. cerevisiae HAP complex, nuclear enrichment of Hap3 and HapX in the *MAT*α strain background disappeared when *HAP2* was deleted (Fig. 5C), indicating that nuclear localization of the Hap2/3/5 complex depends on the NLS of Hap2. In contrast, deletion of *HAP5* did not abolish the nuclear enrichment of HapX in the *MAT*α strain background (Fig. 5D), indicating that Hap2, but not Hap5, is required for the nuclear localization of the HAP complex.

Notably, we found that cellular localization of the HAP complex changed dynamically during mating. When *MAT*α *hap2*Δ::*HAP2-mCherry* and *MAT***a** *hap2*Δ::*HAP2-GFP* cells were mated, a portion of the HAP complex appeared to be localized to the cellular membrane. Similar findings were obtained during mating between *MAT*α *hap3*Δ::*HAP3-mCherry* and *MAT***a** *hap3*Δ::*HAP3-GFP* cells, *MAT*α *hap5*Δ::*HAP5-mCherry* and *MAT***a** *hap5*Δ::*HAP5-GFP* cells, and *MAT*α *hapX*Δ::*HAPX-mCherry* and *MAT***a** *hapX*Δ::*HAPX-GFP* cells (Fig. 5E). All these data imply that the HAP complex primarily acts as a transcriptional repressor and is therefore relocalized from the nucleus to the cellular membrane during mating in C. neoformans.

### Role of the HAP complex in the localization of pheromone receptors and transporters during mating.

**FIG 6:**
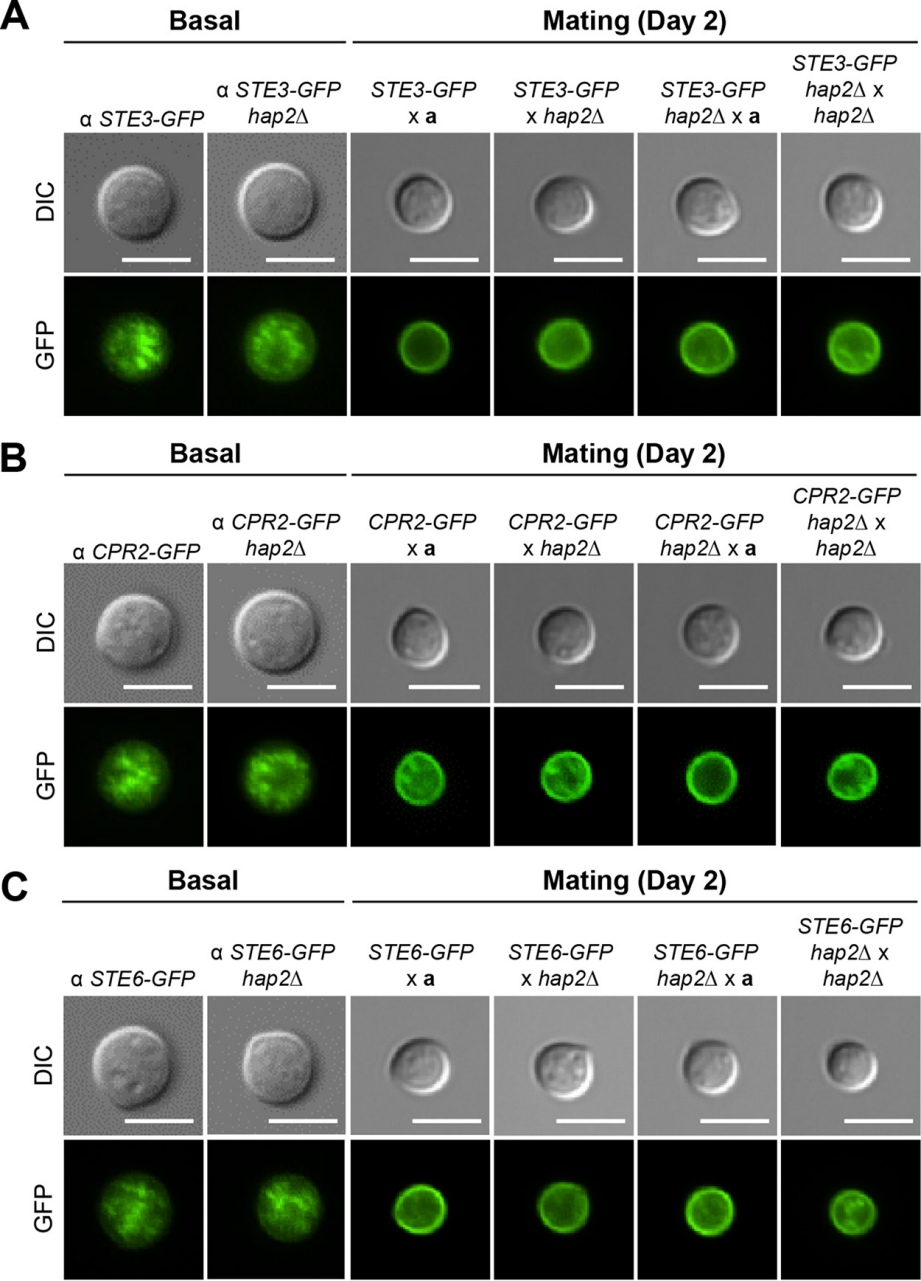
Role of the HAP complex in the localization of the pheromone receptors and transporters during mating. The strains were cultured in liquid YPD medium at 30°C overnight and fixed with formaldehyde. Bars, 5 μm. (A) The localization of Ste3 (mating pheromone receptor) was visualized under basal conditions using α *STE3-GFP* (YSB2864) and α *STE3-GFP hap2*Δ (YSB7815) strains. The localization of Ste3 was also visualized in the dark, under mating conditions, by coculturing the cells harboring α *STE3-GFP* or α *STE3-GFP hap2*Δ with the *MAT***a** YL99**a** or *hap2*Δ strain (YSB5081) for the indicated time periods. (B) The localization of Cpr2 (mating pheromone receptor) was visualized under basal conditions using the α *CPR2-GFP* (YSB3000) and α *CPR2-GFP hap2*Δ (YSB7686) strains. The localization of Cpr2 was also visualized under mating conditions using the method described above. (C) The localization of Ste6 (mating pheromone transporter) was visualized under basal conditions using α *STE6-GFP* (YSB2619) and α *STE6-GFP hap2*Δ (YSB7689) strains. The localization of Ste6 was also visualized under mating conditions using the method described above.

The finding that a portion of the HAP complex can relocalize to the cell membrane during mating prompted us to determine whether the HAP complex affects the membrane localization of mating pheromone transporters (Ste6) and pheromone receptors (Ste3 and Cpr2) during sexual development in C. neoformans. As previously reported, these proteins are localized in the ER during basal conditions, but when the cell is signaled to undergo sexual differentiation, they translocate to the cell membrane (Fig. 6A to C) (49). In the absence of a mating partner, the deletion of *HAP2* did not cause a change in the localization of Ste3, Cpr2, and Ste6, and they remained in a punctate form in the ER. In the presence of an opposite mating partner (YL99**a** or *hap2*Δ), Ste3-GFP, Ste6-GFP, and Cpr2-GFP were translocated to the cell membrane 2 days following mating. To determine the role of Hap2 in the localization of Ste3, Ste6, and Cpr2 during mating, we deleted *HAP2* in genes expressing Ste3-GFP, Ste6-GFP, and Cpr2-GFP. Deletion of *HAP2* led to no changes in the localization of pheromone receptors and transporters. Therefore, it can be deduced that the changes conferred on the development of C. neoformans by the HAP complex are not related to the localization of Ste3, Cpr2, or Ste6 (Fig. 6A to C).

## DISCUSSION

The role of the HAP complex has been extensively studied in eukaryotic organisms ranging from fungi to humans. As both adapting to varying iron levels and the ability to acquire iron are essential for ensuring survival in the host environment, a majority of studies have focused on the role of the HAP complex in iron utilization in pathogenic microorganisms. Studies have supported that the HAP complex plays a critical role in regulating iron homeostasis and pathogenicity of C. neoformans (34, 50). In this study, we demonstrated that the HAP complex also plays a pivotal role in sexual development of C. neoformans by negatively regulating pheromone production and cell fusion events in a Cpk1 MAPK-dependent manner.

At this point, it remains elusive whether the roles of the HAP complex in sexual development and iron utilization are functionally interconnected in C. neoformans. It was previously reported that a GATA-type iron transcription factor, Cir1, has a positive role in mating of C. neoformans (51), which is in stark contrast to the negative role of the HAP complex in mating. Although the HAP complex and Cir1 play coordinated roles in iron utilization and uptake (6), the role of Cir1 in mating appears to be mainly mediated by regulating genes involved in copper uptake, but not those in iron homeostasis (51). However, it remains unknown whether the HAP complex is also involved in regulation of genes involved in copper uptake. It was recently reported that HapX is not likely to directly control copper regulated genes (6). Furthermore, it was also previously reported that the addition of copper, but not iron, induces sexual reproduction of C. neoformans on defined V8 medium (52), implying that the iron utilization pathway may be independent of the sexual development pathway. However, we cannot exclude the possibility that the HAP complex could play an indirect role in copper uptake and metabolism through its role in iron homeostasis. Therefore, the correlation between iron and copper utilization and sexual development with regard to the HAP complex needs to be further studied in the future.

Notably, although the HAP complex plays a conserved role in iron acquisition and utilization as well as sexual development regardless of the mating type, the cellular localization of the HAP complex varies between *MAT*α and *MAT***a** cells. Under nonmating basal conditions, the HAP complex was localized in the nuclei of *MAT*α cells, whereas it was distributed in both the nucleus and cytoplasm in *MAT***a** cells, indicating that the level of the HAP complex observed in the nuclei of *MAT***a** cells may be sufficient for its roles in iron utilization and mating like the nuclear-enriched HAP complex in *MAT*α cells. As the H99 and YL99**a** strains we used are congenic except the *MAT* locus (53), the different localization of the HAP complex may be caused by divergent *MAT*α and *MAT***a** alleles. The *MAT* locus in C. neoformans is unusually large (~120 kb) and encompasses more than 20 genes involved in mating, virulence, and cell viability (54). The presence of mating-type-specific alleles for some genes (e.g., *STE3α* or *STE3***a** and *SXI1α* or *SXI2***a**) or extensive rearrangement of the genes in the *MAT* locus may directly or indirectly contribute to the different cellular localization of the HAP complex between *MAT*α and *MAT***a** cells. This possibility should be further addressed in future studies.

The key finding of this study is that the HAP complex plays a critical role as a transcriptional repressor in the early stage of sexual development of C. neoformans. Our data showed that Hap2/3/5 and HapX play major and minor roles, respectively, in the process. A possible explanation is that the Hap2/3/5 complex without HapX can still bind to the promoter regions of the mating-associated genes and repress them, although the recruitment of HapX may further stabilize the HAP complex. In Aspergillus fumigatus, HapB (an ortholog of Hap2) is the actual CCAAT box-binding subunit, and HapC and HapE (Hap3 and Hap5 orthologs, respectively) subunits recruit and assemble the HapX transcriptional activator with the HapB/C/E heterotrimeric complex (9, 30). Supporting this, our data also showed that Hap2 is the critical DNA binding subunit for the HAP complex in C. neoformans, because the deletion of *HAP2* eliminated the nuclear enrichment of Hap3 and HapX. However, it remains unclear whether Hap5 is necessary for the recruitment of HapX in C. neoformans, because deletion of *HAP5* did not affect the nuclear enrichment of HapX. However, it is still possible that a lack of HapX might decrease the nuclear translocation, DNA-binding activity, and/or stability of the HAP complex in C. neoformans. Otherwise, it is also possible that the transcriptional activator domain of the HapX subunit may independently contribute to the mating repression process. These possibilities should be addressed in future studies.

The role of the HAP complex in fungal development is not confined to C. neoformans. In A. fumigatus, the HAP complex is composed of HapB, HapC, HapE, and HapX (11, 47, 48, 55). Similar to our findings in C. neoformans, deletion of the HAP complex leads to the repression of conidiation in liquid submerged cultures, causing asexual conidiation (56). However, this phenomenon was not observed when the experiment was performed on solid medium—on solid medium, the HAP complex mutants displayed fewer conidia than the wild type. Therefore, it is yet to be determined whether the HAP complex is affected by different culture conditions and whether it plays a conserved role in fungal development.

Here, we provide experimental evidence showing that the HAP complex regulates the sexual differentiation of C. neoformans mainly through the pheromone-responsive Cpk1 MAPK pathway. First, deletion of Hap2/3/5 components displayed an increase in the expression levels of *GPA2*, *CRG1*, *MAT2*, *ZNF2*, and *MFα1*, which are all key signaling components of the Cpk1 MAPK pathway, during the bilateral mating of Hap2/3/5 mutants as opposed to wild-type mating, clearly supporting the repressive role of the HAP complex in sexual differentiation in C. neoformans. Second, ChIP-qPCR analysis clearly showed that the HAP complex could directly bind to the CCAAT motif-containing promoter regions of *CRG1* and *GPA2*. Such binding of the HAP complex to the promoters of development-related genes has been previously shown in other fungi. In A. fumigatus, *hapB* deletion leads to upregulation of key conidiation regulatory genes, including *brlA*, a key regulator of conidiation. It was also reported that the A. fumigatus HAP complex binds to the CCAAT motif in the promoter region of *brlA* to repress its expression (56). Therefore, although the general ability of the HAP complex to bind to other CCAAT motifs of signaling components in the pheromone-responsive MAPK pathway during the developmental cycle remains to be elucidated, this phenomenon may be prevalent in other fungal species.

Another notable finding of this study was that the HAP complex underwent dynamic changes in its cellular localization during mating, and a portion of the HAP complex in the nucleus and cytoplasm appeared to translocate to the cell membrane during mating. Similarly, pheromone receptors (Ste3 and Cpr2) and pheromone transporters (Ste6) also relocalize to the cell membrane during sexual development in C. neoformans (49). However, we found that the deletion of *HAP2* did not affect cellular relocalization, indicating that the two events were unrelated. Membrane localization of the HAP complex has not been reported for other fungi or humans. A similar case was reported for plant cells, where the NF-YC subunit was localized in both the nucleus and the cytoplasmic membrane and possibly in the cell wall (57). However, Li et al. did not investigate the impact of the membrane/cell wall localization of the NF-Y complex on its function. In this study, we could not find any motifs or domains that could facilitate membrane localization of the cryptococcal HAP complex. We speculate that the membrane localization of the HAP complex may contribute to its reduced nuclear localization, which subsequently enhances the mating process in C. neoformans.

In conclusion, the HAP complex plays a critical role in the developmental cycle of C. neoformans by primarily acting as a transcriptional repressor during mating through its association with the Cpk1 MAPK pathway. Despite the phenotypic similarities of this complex in both *MAT*α and *MAT***a**, differences in the localization of the HAP complex exist, which could highlight the differences between the two mating types. Given these findings, this study may prove helpful in elucidating the signaling components associated with the developmental cycle of C. neoformans, as well as unveiling the complexity underlying fungal mating types.

## MATERIALS AND METHODS

### Construction of Cryptococcus neoformans HAP complex mutants and complementation strains.

*HAP2*, *HAP3*, *HAP5*, and *HAPX* were deleted in the C. neoformans serotype A *MAT*α wild-type strain (H99) and *MAT***a** strain (YL99**a**) backgrounds through homologous recombination using gene disruption cassettes containing the nourseothricin resistance marker (*NAT*) and neomycin resistance marker (*NEO*), respectively. Mutants were constructed as previously described (58). The correct genotype of each screened transformant was verified using Southern blot analysis with a gene-specific probe (Fig. S1). The corresponding complemented strains, in which each wild-type allele was reintegrated into its native locus, were constructed using the Gibson assembly method. First, each full-length gene fragment was amplified via Phusion PCR using genomic DNA of the H99 or YL99**a** strain as the template. The amplified fragments of the *MAT*α HAP genes were cloned into the pNEO_mCherry plasmid, and the amplified fragments of the *MAT***a** HAP genes were cloned into the pHYG_GFP plasmid. After confirmation of the integration of the target genes into their respective plasmids through enzyme digestion and sequencing, targeted reintegration into the native locus was conducted. Plasmids containing the HAP genes were linearized by enzyme digestion (Fig. S2) and introduced into each mutant strain via biolistic transformation. The correct genotype for the complemented strain was confirmed using diagnostic PCR.

### Immunoblotting and construction of the Hap2-4×FLAG strain.

The *hap2*Δ::*HAP2-4×FLAG* strain was constructed using the Gibson assembly method. The *HAP2* gene in *MAT*α was amplified using the primers listed in Table S2 and was integrated into the pNEO_4×FLAG plasmid. Integration was confirmed via enzymatic digestion and sequencing. Plasmid was linearized with the enzyme AatII for biolistic transformation into the *hap2*Δ (YSB1104) mutant, and reintegration into its native locus was confirmed using diagnostic PCR. To confirm the constructed strain using immunoblotting, the Hap2-4×FLAG strain was incubated in yeast extract-peptone-dextrose (YPD) broth overnight at 30°C. The overnight culture was inoculated into 50 mL of fresh YPD broth and incubated at 30°C until the optical density at 600 nm (OD_600_) reached approximately 0.8. Immunoblotting with anti-FLAG (Santa Cruz Biotechnology, USA) was conducted as described previously (59).

10.1128/msphere.00170-22.9 TABLE S1 Cryptococcus neoformans strains used in this study. Download Table S1, DOCX file, 0.03 MB. Copyright © 2022 Kim and Bahn. 2022 Kim and Bahn. https://creativecommons.org/licenses/by/4.0/ This content is distributed under the terms of the Creative Commons Attribution 4.0 International license.

10.1128/msphere.00170-22.10 TABLE S2 List of primers used in this study. Download Table S2, DOCX file, 0.02 MB. Copyright © 2022 Kim and Bahn. 2022 Kim and Bahn. https://creativecommons.org/licenses/by/4.0/ This content is distributed under the terms of the Creative Commons Attribution 4.0 International license.

### Mating assay.

To examine unilateral and bilateral mating efficiencies, each HAP complex mutant constructed in the *MAT*α H99 strain and *MAT***a** YL99**a** background were separately cultured in YPD medium for 16 h at 30°C and washed twice with phosphate-buffered saline (PBS). For unilateral mating, HAP mutant cells of one mating type were mixed at equal concentrations (10^7^ cells/ml) with wild-type cells of the opposite mating type. For bilateral mating, HAP mutant cells of one mating type were mixed at equal concentrations (10^7^ cells/ml) with the same HAP mutant of the opposite mating type. These mixtures were spotted onto V8 mating medium (pH 5) or Murashige and Skoog medium (pH 5.8) and incubated at 25°C in the dark for 7 to 14 days. Filamentous growth and sporulation were observed and photographed using a differential interference contrast (DIC) microscope (BX51; Olympus, Tokyo, Japan) and an Olympus BX51 microscope equipped with a SPOT Insight digital camera (Diagnostic Instruments, Inc.).

### Cell fusion assay.

To examine the cell fusion efficiency, *NAT-*marked *MAT*α HAP mutants and *NEO-*marked *MAT***a** HAP mutants were cultured in YPD medium for 16 h at 30°C, washed twice with PBS, mixed at equal concentrations (10^7^ cells/ml), spotted onto V8 mating medium, and incubated for 24 h at 25°C in the dark. The cells were scraped, resuspended, and 100-fold diluted in distilled water (dH_2_O), and 200 μL of each sample was spread onto YPD medium containing both nourseothricin (100 μg/mL) and neomycin (50 μg/mL) to ensure that only the cells that had undergone cell fusion could grow. The plates were incubated at 25°C in the dark for 4 to 5 days. The *NAT/NEO-*positive dikaryotic cells were counted using an automated bacterial colony counter (aCOLyte 3; Synbiosis Ltd., UK).

### Expression analysis using Northern blotting and qRT-PCR.

To monitor the expression levels of the genes involved in mating, we performed Northern blot analysis and qRT-PCR. To extract RNA, the H99, YL99**a**, and mutant strains were incubated in liquid YPD medium for 16 h at 30°C. The cell culture was washed three times with PBS and prepared for unilateral and bilateral mating using a mixture of equal cell concentrations (10^8^ cells/ml). The cells were spread onto V8 medium for 6 and 12 h, scraped, and lyophilized overnight. For the zero-time control, the *MAT*α and *MAT***a** cells were freshly mixed and collected, washed, and lyophilized overnight. Total RNA was extracted from each sample using a commercial RNA extraction kit (easy-BLUE; iNtRON Biotechnology, South Korea), and cDNA was synthesized using reverse transcriptase (RTase) (Thermo Scientific, Waltham, MA). To monitor gene expression levels, we performed qRT-PCR with gene-specific primer pairs using a CFX96 Touch real-time PCR detection system (Bio-Rad). For Northern blotting analysis, the membrane was hybridized using a radioactively labeled probe generated from gene-specific primers, as previously described (60).

### ChIP-qPCR.

Hap2-4**×**FLAG or HapX-FLAG strains were grown in 50 ml of YPD broth overnight at 30°C and subcultured to an OD_600_ of 0.8 at 30°C. Cells were harvested by centrifugation and washed twice with PBS. Chromatin immunoprecipitation (ChIP) was performed as previously described (61). To monitor the binding of Hap2 and HapX to the *CRG1* and *GPA2* promoters, we employed qRT-PCR. Whole-cell extracts (WCE) and IP samples were used as templates for qRT-PCR. Primers were designed to encompass the regions of *CRG1* and *GPA2*, where Hap2 and HapX were assumed to bind owing to their respective consensus sequences.

10.1128/msphere.00170-22.8 FIG S8 Construction of *STE3-GFP hap2*Δ, *CPR2-GFP hap2*Δ, and *STE6-GFP hap2*Δ mutant strains. (A) Diagram of the *HAP2* gene disruption strategy in the *MAT*α *STE3-GFP, MAT*α *CPR2-GFP*, and *MAT*α *STE6-GFP* strains. (B to D) Confirmation of *STE3-GFP hap2*Δ, *CPR2-GFP hap2*Δ, and *STE6-GFP hap2*Δ gene disruption using Southern blot analysis. Download FIG S8, PDF file, 0.3 MB. Copyright © 2022 Kim and Bahn. 2022 Kim and Bahn. https://creativecommons.org/licenses/by/4.0/ This content is distributed under the terms of the Creative Commons Attribution 4.0 International license.
