# Supplementary material for: Role of the Heme Activator Protein Complex in the Sexual Development of Cryptococcus neoformans
Source: mSphere. 2022 May 31;7(3):e00170-22. doi: 10.1128/msphere.00170-22 (PMC9241503; doi:10.1128/msphere.00170-22)
Supplement: FIG S1 [file msphere.00170-22-sf001.pdf]

Fig S1

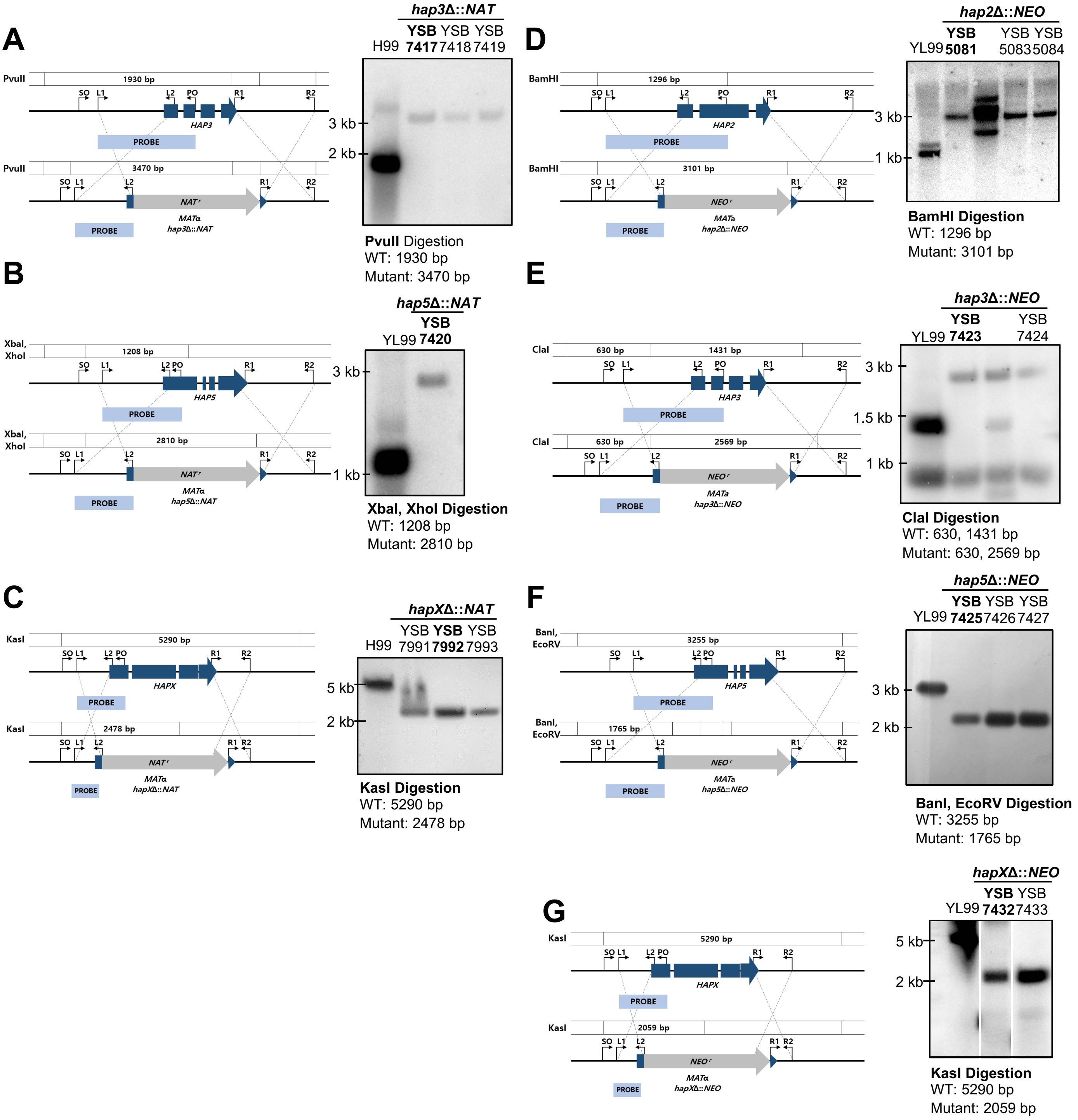

Fig. S1. Disruption of *Cryptococcus neoformans* HAP complex in serotype A *MAT*α H99 and *MAT*a YL99

Disruption of the *HAP3*, *HAP5*, and *HAPX* genes in the *MAT*α H99 strain (A-C) and *HAP2*, *HAP3*, *HAP5*, and *HAPX* genes in the *MAT***a** YL99 strain (D-G). The correct gene disruption was verified by Southern blot analysis using genomic DNAs digested with the indicated restriction enzymes. The representative strains used in this study are shown in bold.
