# Supplementary material for: Role of the Heme Activator Protein Complex in the Sexual Development of Cryptococcus neoformans
Source: mSphere. 2022 May 31;7(3):e00170-22. doi: 10.1128/msphere.00170-22 (PMC9241503; doi:10.1128/msphere.00170-22)
Supplement: FIG S2 [file msphere.00170-22-sf002.pdf]

Fig S2

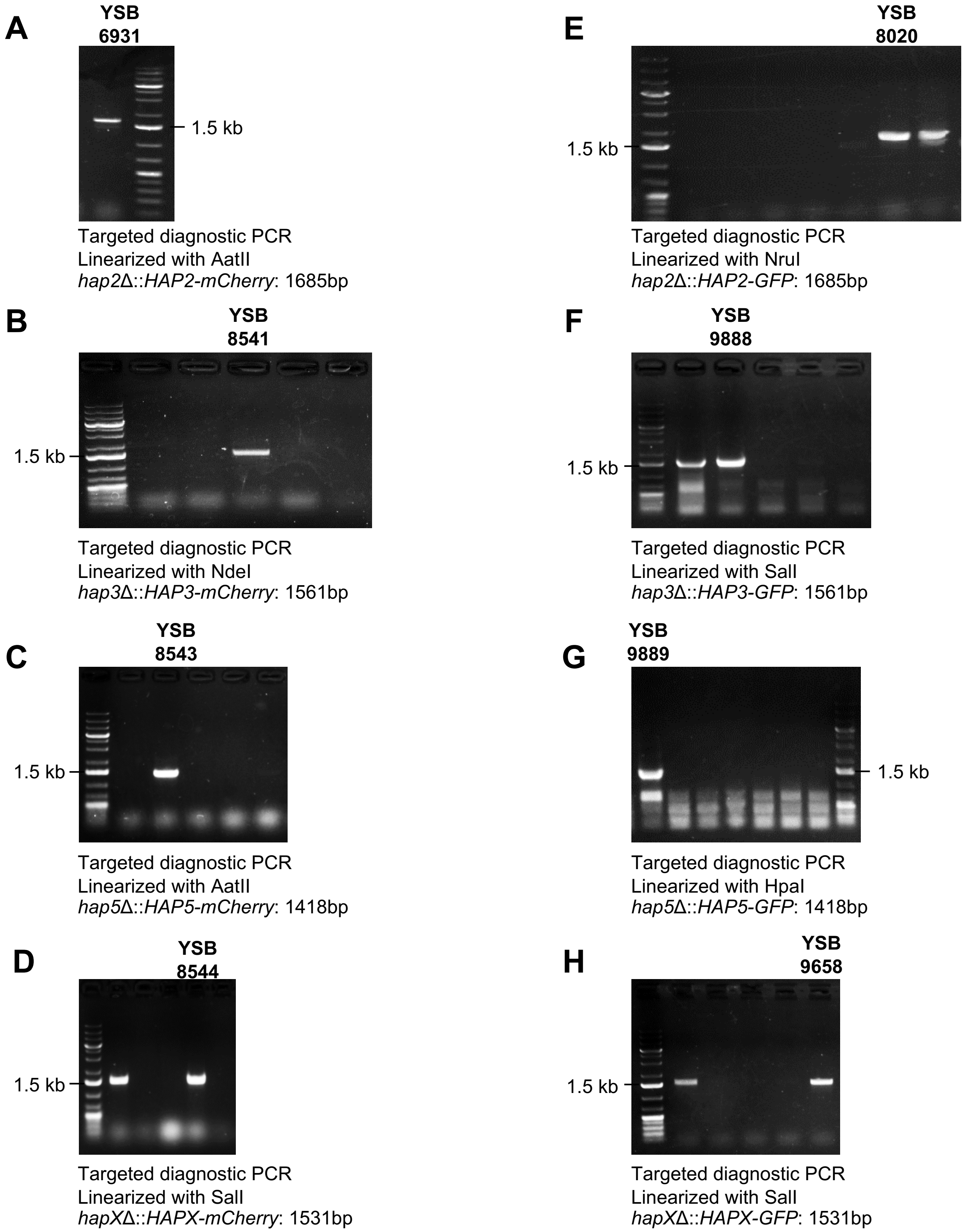

Fig. S2. Construction of HAP complex-complemented strains in serotype A *MAT*α H99 and *MAT*a YL99

The complemented strains were confirmed via targeted diagnostic PCR, using the specific primer pairs listed in Table S2.
