# Supplementary material for: Role of the Heme Activator Protein Complex in the Sexual Development of Cryptococcus neoformans
Source: mSphere. 2022 May 31;7(3):e00170-22. doi: 10.1128/msphere.00170-22 (PMC9241503; doi:10.1128/msphere.00170-22)
Supplement: FIG S3 [file msphere.00170-22-sf003.pdf]

Fig S3

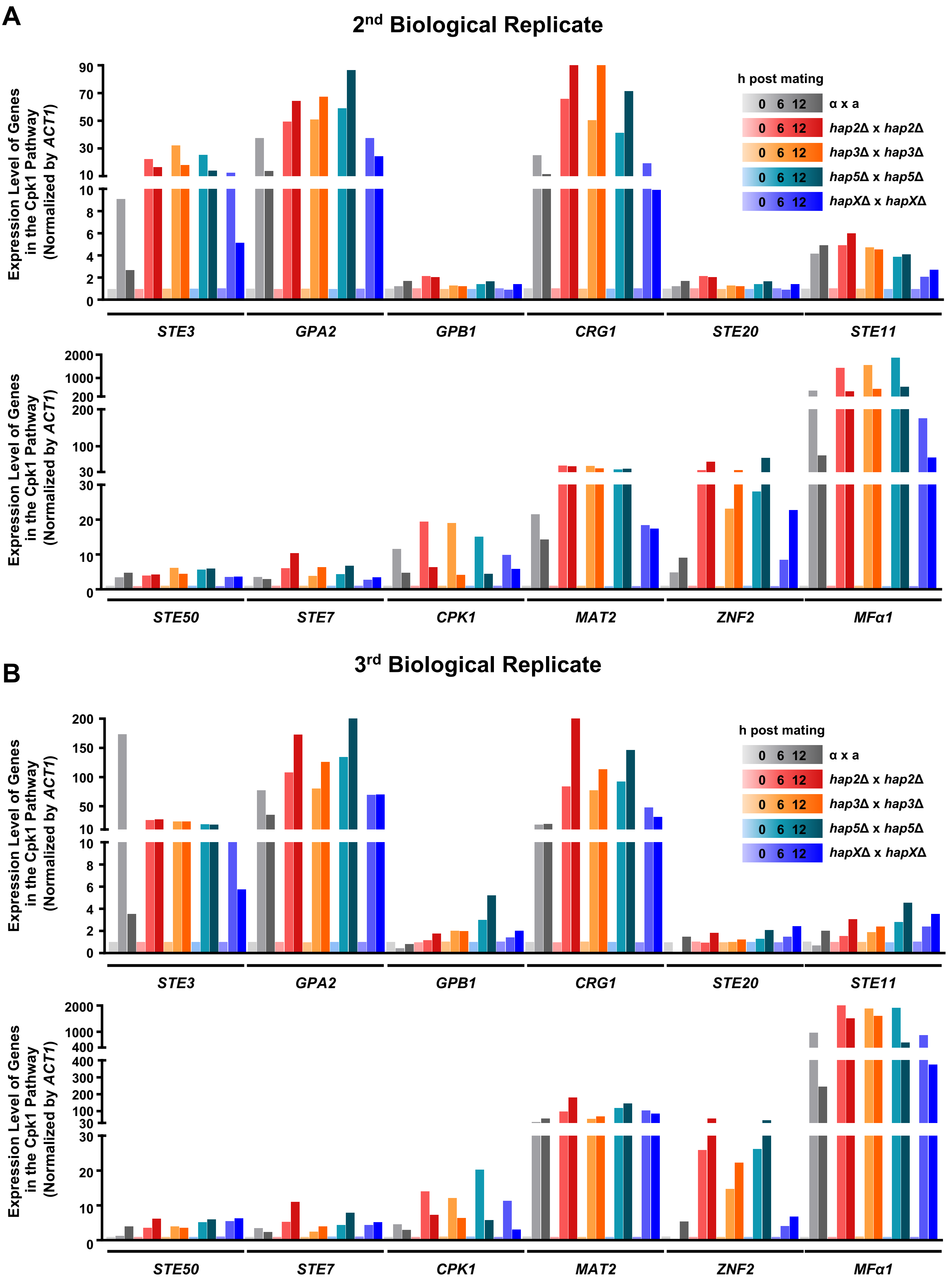
A
2nd Biological Replicate
Expression Level of Genes in the Cpk1 Pathway (Normalized by ACT1)
h post mating
0 6 12 α x a
0 6 12 hap2Δ x hap2Δ
0 6 12 hap3Δ x hap3Δ
0 6 12 hap5Δ x hap5Δ
0 6 12 hapXΔ x hapXΔ
STE3
GPA2
GPB1
CRG1
STE20
STE11
STE50
STE7
CPK1
MAT2
ZNF2
MFα1
B
3rd Biological Replicate
Expression Level of Genes in the Cpk1 Pathway (Normalized by ACT1)
h post mating
0 6 12 α x a
0 6 12 hap2Δ x hap2Δ
0 6 12 hap3Δ x hap3Δ
0 6 12 hap5Δ x hap5Δ
0 6 12 hapXΔ x hapXΔ
STE3
GPA2
GPB1
CRG1
STE20
STE11
STE50
STE7
CPK1
MAT2
ZNF2
MFα1

Fig. S3. Expression levels of genes in the Cpk1 pathway during bilateral mating of HAP complex mutant strains

qRT-PCR was used to monitor the expression levels of various genes in the Cpk1 pathway using RNA isolated from co-cultures of HAP mutants on V8 medium. RNA was isolated from co-cultures at 0, 6, and 12 h following mating, and the expression levels of these genes were normalized to the expression levels of the housekeeping gene *ACT1*. The expression levels of these genes at 0 h were set at 1. (A) Results for the second biological replicate and (B) third biological replicate of this experiment.
