# Supplementary material for: Role of the Heme Activator Protein Complex in the Sexual Development of Cryptococcus neoformans
Source: mSphere. 2022 May 31;7(3):e00170-22. doi: 10.1128/msphere.00170-22 (PMC9241503; doi:10.1128/msphere.00170-22)
Supplement: FIG S4 [file msphere.00170-22-sf004.pdf]

Fig S4

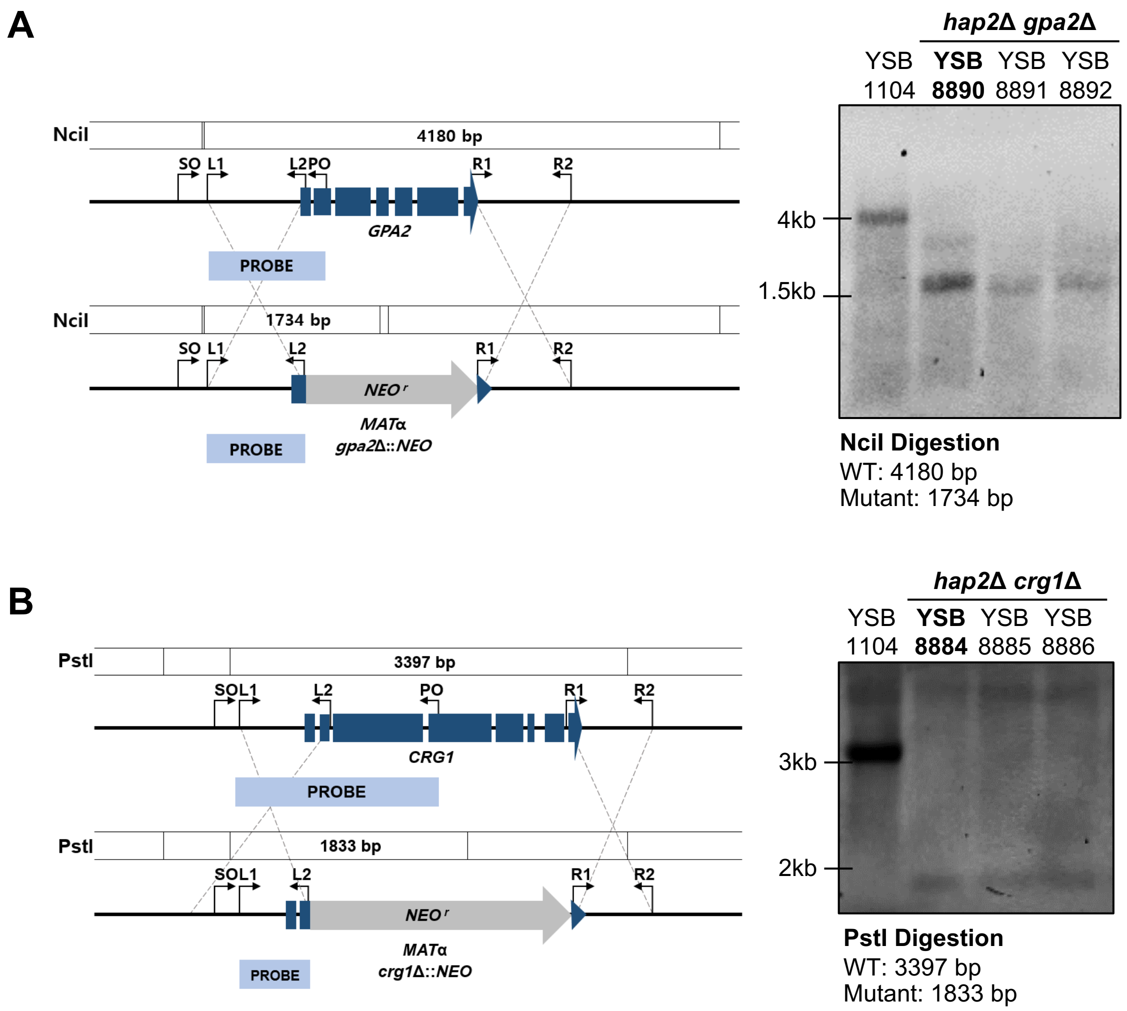

Fig. S4. The construction of *hap2Δ gpa2Δ* and *hap2Δ crg1Δ* mutant strains.

(A) Schematic representation of the *GPA2* disruption strategy and confirmation of the deletion of the *GPA2* gene in the *MATα hap2Δ* (YSB1104) mutant strain through Southern blot analysis. (B) Schematic representation of *CRG1* disruption strategy and confirmation of the deletion of the *CRG1* gene in *hap2Δ* mutant strain through Southern blot analysis.
