# Supplementary material for: Role of the Heme Activator Protein Complex in the Sexual Development of Cryptococcus neoformans
Source: mSphere. 2022 May 31;7(3):e00170-22. doi: 10.1128/msphere.00170-22 (PMC9241503; doi:10.1128/msphere.00170-22)
Supplement: FIG S5 [file msphere.00170-22-sf005.pdf]

Fig S5

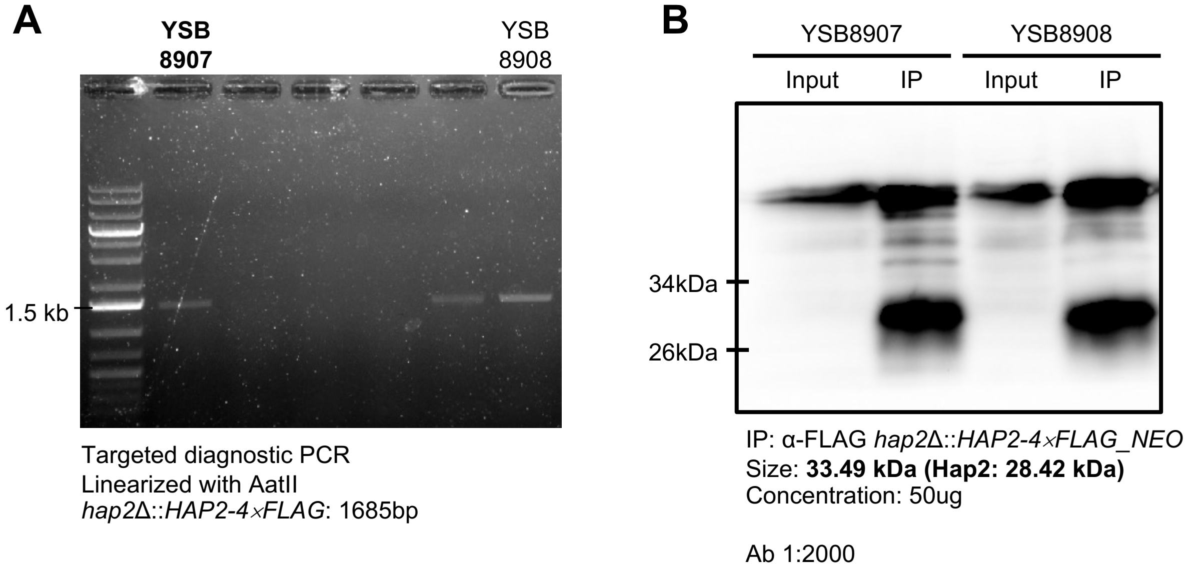

Fig. S5. Construction of FLAG-tagged Hap2 strain

(A) The targeted reintegration of the pHAP2-4×FLAG plasmid to *MAT*α *hap2*Δ (YSB1104) was confirmed using diagnostic PCR. (B) The functionality of the constructed strain was tested using immunoprecipitation of the Hap2-4×FLAG protein with an anti-FLAG antibody (IP: α-FLAG).
