# Supplementary material for: Role of the Heme Activator Protein Complex in the Sexual Development of Cryptococcus neoformans
Source: mSphere. 2022 May 31;7(3):e00170-22. doi: 10.1128/msphere.00170-22 (PMC9241503; doi:10.1128/msphere.00170-22)
Supplement: FIG S6 [file msphere.00170-22-sf006.pdf]

Fig S6

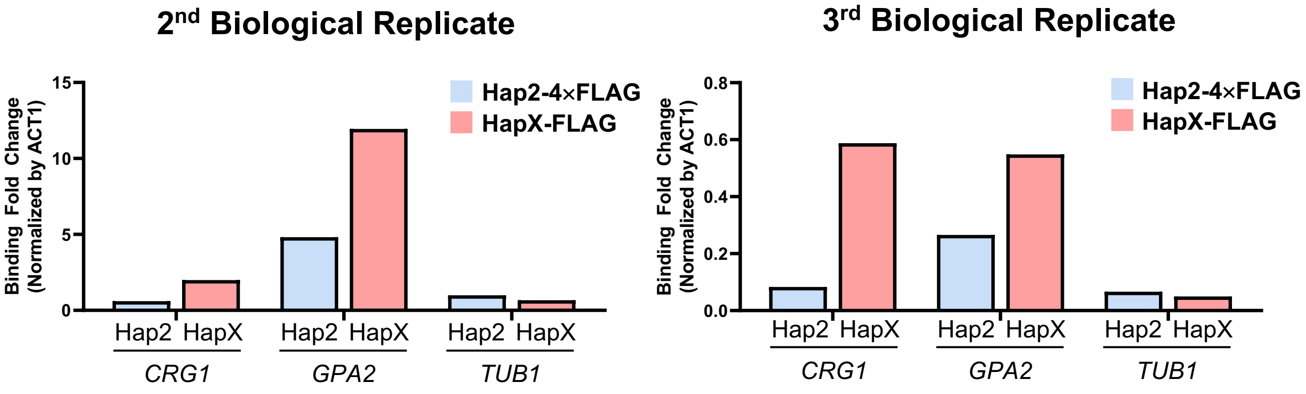

Fig S6. Binding of Hap2 and HapX to *CRG1* and *GPA2* Promoters

Three biological replicates of ChIP-qPCR were used to examine the binding of the Hap2-4×FLAG and HapX-FLAG strains to the promoters of *CRG1*, *GPA2*, and *TUB1*. ChIP-qPCR was performed under basal conditions for strains *MAT*α *hap2*Δ*::HAP2-4×FLAG* (YSB8907) and *MAT*α *hapX*Δ*::HAPX-FLAG* (1). The fold changes in binding of Hap2 and HapX to *CRG1, GPA2,* and *TUB1* were calculated as follows: $2^{-[IP(Gene\text{-}Actin) - WCE\ (Gene\text{-}Actin)]}$.

1. Do E, Cho YJ, Kim D, Kronstad JW, Jung WH. 2020. A transcriptional regulatory map of iron homeostasis reveals a new control circuit for capsule formation in *Cryptococcus neoformans*. Genetics 215:1171-1189.
