# Supplementary material for: Role of the Heme Activator Protein Complex in the Sexual Development of Cryptococcus neoformans
Source: mSphere. 2022 May 31;7(3):e00170-22. doi: 10.1128/msphere.00170-22 (PMC9241503; doi:10.1128/msphere.00170-22)
Supplement: FIG S7 [file msphere.00170-22-sf007.pdf]

Fig S7

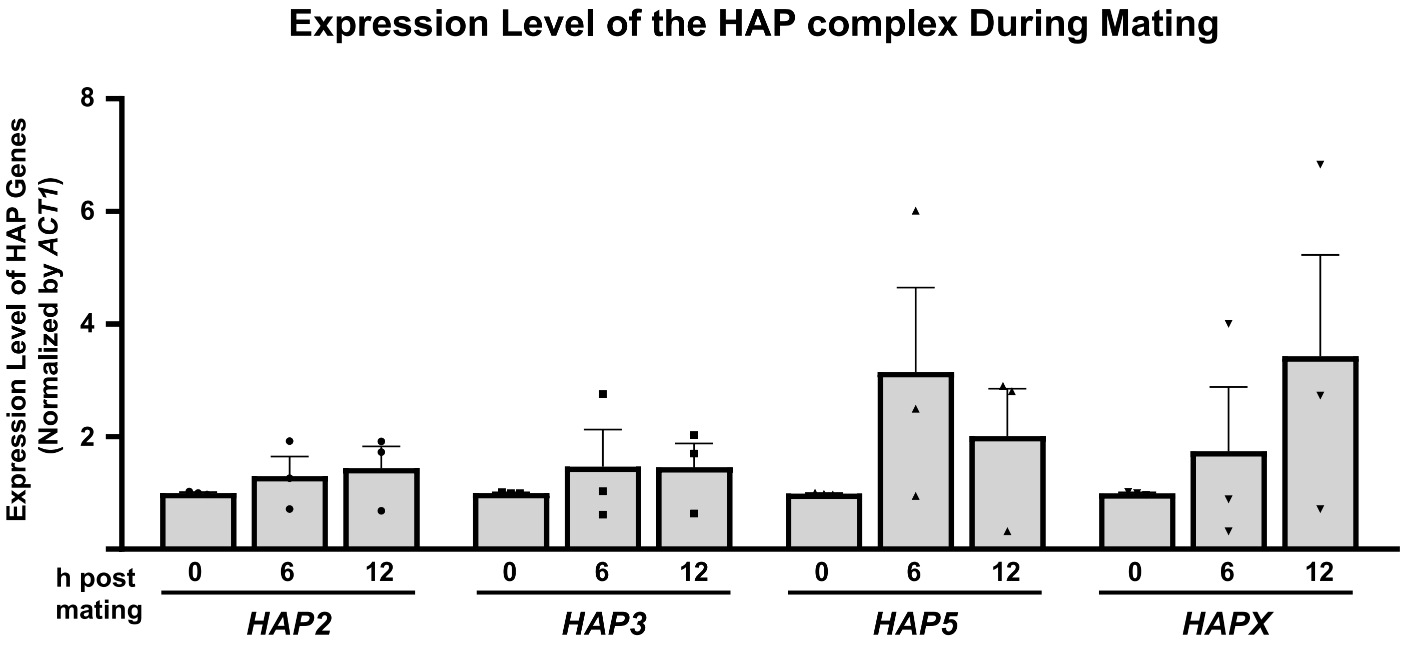

Fig S7. Transcriptional regulation of the HAP complex during mating.

The α (H99) and **a** (YL99) strains were cultured in liquid YPD medium at 30°C overnight. The cells were co-incubated for 0, 6, and 12 h on V8 medium and scraped at the corresponding time points. The expression of each HAP complex gene was examined using qRT-PCR by normalizing the gene expression levels with *ACT1*.
