# Supplementary material for: Role of the Heme Activator Protein Complex in the Sexual Development of Cryptococcus neoformans
Source: mSphere. 2022 May 31;7(3):e00170-22. doi: 10.1128/msphere.00170-22 (PMC9241503; doi:10.1128/msphere.00170-22)
Supplement: TABLE S1 [file msphere.00170-22-st001.docx]

**Supplementary Table 1. List of strains used in this study.**

| Strain | Genotype | Parent | Reference |
| --- | --- | --- | --- |
| H99 | *MAT*α |  | (1) |
| YL99 | *MAT***a** |  | (2) |
| YSB1104 | *MAT*α *hap2*Δ*::NAT-STM #123* | H99 | (3) |
| YSB8026 | *MAT*α *hapX*Δ::*NAT* *HAPX-*FLAG*-NEO* |  | (4) |
| YSB2864 | *MAT*α *STE3::GFP* | H99 | (5) |
| YSB3000 | *MAT*α *CPR2::GFP* | H99 | (5) |
| YSB2619 | *MAT*α *STE6::GFP* | H99 | (5) |
| YSB5081 | *MAT***a** *hap2*Δ::*NEO* | YL99 | This study |
| YSB7417 | *MAT*α *hap3*Δ::*NAT-STM* #231 | H99 | This study |
| YSB7423 | *MAT***a** *hap3*Δ::*NEO* | YL99 | This study |
| YSB7420 | *MAT*α *hap5*Δ::*NAT-STM* #232 | H99 | This study |
| YSB7425 | *MAT***a** *hap5*Δ::*NEO* | YL99 | This study |
| YSB7992 | *MAT*α *hapX*Δ::*NAT-STM* #240 | H99 | This study |
| YSB7432 | *MAT***a** *hapX*Δ::*NEO* | YL99 | This study |
| YSB6931 | *MAT*α *hap2*Δ::*HAP2-mCherry* | YSB1104 | This study |
| YSB8020 | *MAT***a** *hap2*Δ*::HAP2-GFP* | YSB5081 | This study |
| YSB8541 | *MAT*α *hap3*Δ::*HAP3-mCherry* | YSB7417 | This study |
| YSB9888 | *MAT***a** *hap3*Δ::*HAP3*-*GFP* | YSB7423 | This study |
| YSB8543 | *MAT*α *hap5*Δ::*HAP5-mCherry* | YSB7420 | This study |
| YSB9889 | *MAT***a** *hap5*Δ::*HAP5*-*GFP* | YSB7425 | This study |
| YSB8544 | *MAT*α *hapX*Δ::*HAPX-mCherry* | YSB7992 | This study |
| YSB9658 | *MAT***a** *hapX*Δ::*HAPX*-*GFP* | YSB7432 | This study |
| YSB7815 | *MAT*α *STE3*::*GFP* *hap2*Δ::*NAT-STM* #123 | YSB2864 | This study |
| YSB7689 | *MAT*α *STE6*:*GFP* *hap2*Δ::*NAT-STM* #123 | YSB2619 | This study |
| YSB7686 | *MAT*α *CPR2*::*GFP* *hap2*Δ::*NAT-STM* #123 | YSB3000 | This study |
| YSB8884 | *MAT*α *hap2*Δ::*NAT-STM* #123 *crg1*Δ::*NEO* | YSB1104 | This study |
| YSB8890 | *MAT*α *hap2*Δ::*NAT-STM* #123 *gpa2*Δ::*NEO* | YSB1104 | This study |
| YSB9660 | *MAT*α *hap3*Δ::*HAP3-mCherry* *hap2*Δ::*HYG* | YSB8541 | This study |
| YSB9890 | *MAT*α *hapX*Δ::*HAPX-mCherry* *hap2*Δ::*HYG* | YSB8544 | This study |
| YSB9891 | *MAT*α *hapX*Δ::*HAPX-mCherry* *hap5*Δ::*HYG* | YSB8544 | This study |
| YSB8907 | *MAT*α *hap2*Δ*::HAP2*-4xFLAG | YSB1104 | This study |

1. Perfect JR, Ketabchi N, Cox GM, Ingram CW, Beiser CL. 1993. Karyotyping of *Cryptococcus neoformans* as an epidemiological tool. J Clin Microbiol 31:3305-9.

2. Semighini CP, Averette AF, Perfect JR, Heitman J. 2011. Deletion of *Cryptococcus neoformans* AIF ortholog promotes chromosome aneuploidy and fluconazole-resistance in a metacaspase-independent manner. PLoS Pathog 7:e1002364.

3. Jung KW, Yang DH, Maeng S, Lee KT, So YS, Hong J, Choi J, Byun HJ, Kim H, Bang S, Song MH, Lee JW, Kim MS, Kim SY, Ji JH, Park G, Kwon H, Cha S, Meyers GL, Wang LL, Jang J, Janbon G, Adedoyin G, Kim T, Averette AK, Heitman J, Cheong E, Lee YH, Lee YW, Bahn YS. 2015. Systematic functional profiling of transcription factor networks in *Cryptococcus neoformans*. Nat Commun 6:6757.

4. Do E, Cho YJ, Kim D, Kronstad JW, Jung WH. 2020. A transcriptional regulatory map of iron homeostasis reveals a new control circuit for capsule formation in *Cryptococcus neoformans*. Genetics 215:1171-1189.

5. Jung KW, So YS, Bahn YS. 2016. Unique roles of the unfolded protein response pathway in fungal development and differentiation. Sci Rep 6:33413.
