# Supplementary material for: Role of the Heme Activator Protein Complex in the Sexual Development of Cryptococcus neoformans
Source: mSphere. 2022 May 31;7(3):e00170-22. doi: 10.1128/msphere.00170-22 (PMC9241503; doi:10.1128/msphere.00170-22)
Supplement: TABLE S2 [file msphere.00170-22-st002.docx]

**Supplementary Table 2. List of primers used in this study.**

| Primer Name | Sequence (5' to 3') | Comments |
| --- | --- | --- |
| B79 | TGTGGATGCTGGCGGAGGATA | Screening Primer |
| B1026 | GTAAAACGACGGCCAGTGAAT | M13 Forward Primer |
| B1027 | CAGGAAACAGCTATGACCATG | M13 Reverse Primer |
| B354 | GCATGCAGGATTCGAGTG | *HYG* Forward Primer |
| B1966 | GGAGCCATGAAGATCCTGA | *HYG* Reverse Primer |
| B1454 | AAGGTGTTCCCCGACGACGAATCG | *NAT* PCR Primer SM1 |
| B1455 | AACTCCGTCGCGAGCCCCATCAAC | *NAT* PCR Primer SM2 |
| B1886 | TGGAAGAGATGGATGTGC | *NEO* PCR Primer SM1 |
| B1887 | ATTGTCTGTTGTGCCCAG | *NEO* PCR Primer SM2 |
| B5751 | CGAAGAATCTCGTGCTTTC | *HYG* PCR Primer SM1 |
| B5752 | ATTGACCGATTCCTTGCG | *HYG* PCR Primer SM2 |
| B2248 | CAACCTTCAAAGTCTTGCC | *HAP2* PCR Primer L1 |
| B2249 | TCACTGGCCGTCGTTTTACAACGAGGAGTCATACTGTGC | *HAP2* PCR Primer L2 |
| B2250 | CATGGTCATAGCTGTTTCCTGAAAGGCGTCAAAGGGCGAAG | *HAP2* PCR Primer R1 |
| B2251 | GGTTTGGAGCGTATTGATTG | *HAP2* PCR Primer R2 |
| B2252 | AAGAGCGGCATTGAATCGG | *HAP2* PCR Primer SO |
| B2253 | GCGTCACTTTGGACATACTC | *HAP2* PCR Primer PO/ *HAP2* qRT Primer qRT2 |
| B10619 | CCTTTATCCCTTTGTCCG | *HAP3* PCR Primer L1 |
| B10620 | TCACTGGCCGTCGTTTTACCTGTAAATGGTTTTGAGGGC | *HAP3* PCR Primer L2 |
| B10621 | CATGGTCATAGCTGTTTCCTGAAGTGATGACGCACAAGG | *HAP3* PCR Primer R1 |
| B10622 | GACCGAGGAAGGTTTGAAC | *HAP3* PCR Primer R2 |
| B10623 | CACCAATGGCAGAAAGAG | *HAP3* PCR Primer SO |
| B10624 | CGATAGTGTAATGGAGGAAGAG | *HAP3* PCR Primer PO |
| B10625 | CCTCAAGAATGATTACGAGAGC | *HAP5* PCR Primer L1 |
| B10626 | TCACTGGCCGTCGTTTTACTGCTGTTTGAGTTTGAGGC | *HAP5* PCR Primer L2 |
| B10627 | CATGGTCATAGCTGTTTCCTGAGGAAGAGGAAGGTGATTTG | *HAP5* PCR Primer R1 |
| B10628 | AACAAACGCCATCTCCTG | *HAP5* PCR Primer R2 |
| B10629 | AACGCTGACACATTCTGAAG | *HAP5* PCR Primer SO |
| B10630 | GTTGGTGGTTGTGGTTTTG | *HAP5* PCR Primer PO |
| B10631 | CCTACCTCAACGGCTATTATC | *HAPX* PCR Primer L1 |
| B10632 | TCACTGGCCGTCGTTTTACTGGGCGAGATTGAATACG | *HAPX* PCR Primer L2 |
| B10633 | CATGGTCATAGCTGTTTCCTGTTATTGGACGAGGCAACG | *HAPX* PCR Primer R1 |
| B10634 | CCAGGTTCCAAATAATCTGC | *HAPX* PCR Primer R2 |
| B10635 | CTCAACTGAAAGCAACCTTG | *HAPX* PCR Primer SO |
| B10636 | TCGGTGTAACAGATTTGGC | *HAPX* PCR Primer PO |
| B12308 | GAGTAACAAAGCGAGCATTC | *CRG1* PCR Primer L1 |
| B12309 | TCACTGGCCGTCGTTTTACTGAAACTGTTGGGATAGGC | *CRG1* PCR Primer L2 |
| B12310 | CATGGTCATAGCTGTTTCCTGGCTCTTACCTCACAATCTCTCAAG | *CRG1* PCR Primer R1 |
| B12311 | GTTGTCTTCCTCGCTTCTC | *CRG1* PCR Primer R2 |
| B12312 | TCCCTGTATCCCTTTTTACG | *CRG1* PCR Primer SO |
| B12314 | ATGGAGACCTTTGGGAGTC | *CRG1* PCR Primer PO |
| B12316 | AACGCACGCTTGAGTTTG | *GPA2* PCR Primer L1 |
| B12317 | TCACTGGCCGTCGTTTTACCGTCTTCGGTAGATTGAGTGC | *GPA2* PCR Primer L2 |
| B12318 | CATGGTCATAGCTGTTTCCTGGTCTTCTCTAATCCCGTCAATC | *GPA2* PCR Primer R1 |
| B12319 | GACTGAGGAAGACCAAGAGG | *GPA2* PCR Primer R2 |
| B12320 | GCCAATAAAAGCCAGACG | *GPA2* PCR Primer SO |
| B12322 | TGACTTTCCTGATTCACCTG | *GPA2* PCR Primer PO |
| B10219 | GATATCTGTCTCCAAAGTCGACGA | *HAP2* Complemented Strain Fwd |
| B10220 | GCGGCCGCCGCAGAGGCT | *HAP2* Complemented Strain Rev |
| B9508 | TCCTTGGGGGACTTTTTC | *HAP2* Complemented Strain Seq1 |
| B9509 | TTCACTCACCTCACCACAC | *HAP2* Complemented Strain Seq2 |
| B9510 | GACCACGAGTATGTCCAAAG | *HAP2* Complemented Strain Seq3 |
| B10366 | GTA TCA AGT CAA GCC CCA C | *HAP2* Complemented Strain CSO |
| B12776 | ACAGAGCCACCGCCACCTGCGGCCGCTCGCACACCTTGTGCGTC | *HAP3* Complemented Strain Fwd |
| B12777 | CTCTAGATGCATGCTCGAGCGGCCGCGGCATGATCTCCTCATTTTTAATACCAC | *HAP3* Complemented Strain Rev |
| B12887 | TCCTTGAGATGGCGGATAG | *HAP3* Complemented Strain Seq1 |
| B12888 | GGAATGTGTCCAAGAATGC | *HAP3* Complemented Strain Seq2 |
| B12961 | TGACGCTGGTAAATCTGTG | *HAP3* Complemented Strain CSO |
| B12778 | ACAGAGCCACCGCCACCTGCGGCCGCATCCCCCTGTACATACTC | *HAP5* Complemented Strain Fwd |
| B12779 | CTCTAGATGCATGCTCGAGCGGCCGCACATTCTGAAGAAACCGG | *HAP5* Complemented Strain Rev |
| B12889 | AGGAGAGTCTGACAGCGAAG | *HAP5* Complemented Strain Seq1 |
| B12890 | TGGACGAGACAGATGGACAC | *HAP5* Complemented Strain Seq2 |
| B12962 | GCAAAGGAGAGAGTATGGAAG | *HAP5* Complemented Strain CSO |
| B12780 | ACAGAGCCACCGCCACCTGCGGCCGCCGGGATTCGTTCGATTCTTC | *HAPX* Complemented Strain Fwd |
| B12781 | CTCTAGATGCATGCTCGAGCGGCCGCGATGCTGAATGCTTTATATTGTAATTTAC | *HAPX* Complemented Strain Rev |
| B12891 | TTTAGGCGGGGTTAGTTC | *HAPX* Complemented Strain Seq1 |
| B12892 | CCAACTCTTCTTCCTCAAGC | *HAPX* Complemented Strain Seq2 |
| B12893 | CAATAGATAGCCTTGTCCCAG | *HAPX* Complemented Strain Seq3 |
| B12894 | CATTCGGTAAGCAACTCG | *HAPX* Complemented Strain Seq4 |
| B12963 | TGGACAATCAAAGGTGAGTC | *HAPX* Complemented Strain CSO |
| B6381 | CCCAGCCCGGGCCATCGA | *HAP2* qRT Primer qRT1 |
| B14744 | GAAGCGGCTGAAAAGTGTTT | *HAP3* qRT Primer qRT1 |
| B14745 | CACCCTCATAATTGTCGAACC | *HAP3* qRT Primer qRT2 |
| B14746 | TGGACGAGACAGATGGACAC | *HAP5* qRT Primer qRT1 |
| B14747 | CTTCCTCGTCGCTCTTCATC | *HAP5* qRT Primer qRT2 |
| B14748 | AATCGACCTGGAAACGTGAC | *HAPX* qRT Primer qRT1 |
| B14749 | TGGGTACTGCTGTTGACGAG | *HAPX* qRT Primer qRT2 |
| B10947 | GCATCATCCATCCTTTCAG | *STE3* qRT Primer qRT1 |
| B10948 | GACACTCCAAAGAACAGGAAG | *STE3* qRT Primer qRT2 |
| B10952 | TACACCACCAACAAGGTCC | *GPB1* qRT Primer qRT1/ *GPB1* Northern Primer NP1 |
| B10953 | AGGGAGTAGATGGAGCAGAC | *GPB1* qRT Primer qRT2 |
| B10954 | CGAGTTCTTTTGGAAGGC | *GPA2* qRT Primer qRT1/ *GPA2* Northern Primer NP1 |
| B10955 | AACTTGGAAGGGAAGACG | *GPA2* qRT Primer qRT2 |
| B10956 | TACATCTTCTCAGCACGGG | *CRG1* qRT Primer qRT1/ *CRG1* Northern Primer NP1 |
| B10957 | CGAACAAACTGAGCAAGG | *CRG1* qRT Primer qRT2 |
| B7660 | CAACGGTATCACCCAAGAC | *STE20* qRT Primer qRT1 |
| B10951 | CCTCTTCTTCATCTTCTCCTTC | *STE20* qRT Primer qRT2 |
| B10945 | CAAAAACCGTCACAACTACC | *STE11* qRT Primer qRT1 |
| B10946 | CCTAACTGAGACCCTATTGACTTC | *STE11* qRT Primer qRT2 |
| B1555 | CACTCTGAAAGATTTGGCG | *STE50* qRT Primer qRT1 |
| B10944 | CAGGACCGTCAAGTCATTC | *STE50* qRT Primer qRT2 |
| B10949 | TCTCTCGGTATCTCCCTTATC | *STE7* qRT Primer qRT1 |
| B10950 | GGGTCAAAGTCGGAAACAC | *STE7* qRT Primer qRT2 |
| B8557 | CTGCGATTTCGGTCTTGCC | *CPK1* qRT Primer qRT1 |
| B8558 | GATACCACCTTGTAGCGAC | *CPK1* qRT Primer qRT2 |
| B11381 | TGAGGGAGAAAAGGTTCC | *MAT2* qRT Primer qRT1 |
| B11382 | GGAGGAGGCATTGACTTATTC | *MAT2* qRT Primer qRT2 |
| B11060 | TTGTGAACGACCACAACCAC | *ZNF2* qRT Primer qRT1 |
| B11061 | TGCCTTGCAAGATCACTTTTT | *ZNF2* qRT Primer qRT2 |
| B8097 | CGCCTTCACTGCCATCTTC | *MFα1* qRT Primer qRT1 |
| B8098 | ACAAAGGGTCATGCCACCGG | *MFα1* qRT Primer qRT2 |
| B679 | CGCCCTTGCTCCTTCTTCTATG | *ACT1* qRT Primer qRT1 |
| B680 | GACTCGTCGTATTCGCTCTTCG | *ACT1* qRT Primer qRT2 |
| B4790 | CTTCTACCTCTGCCTCTTCAC | *STE3* Northern Primer NP1 |
| B4791 | TGTAGAGTCTTTCCAGTCCG | *STE3* Northern Primer NP2 |
| B14594 | AGCTATCCCAGCTGCCTGTA | *GPB1* Northern Primer NP2 |
| B14595 | GACGAACTTCAGAGCCTTGG | *GPA2* Northern Primer NP2 |
| B14596 | CCTTCACTCCAGCTTCCTTG | *CRG1* Northern Primer NP2 |
| B6055 | AGGGACAGTGACCAAAGTG | *STE7* Northern Primer NP1 |
| B6056 | TCTGACTGACACCGTTACG | *STE7* Northern Primer NP2 |
| B14597 | GATGGCCAGTAGGGAGATGA | *STE20* Northern Primer NP1 |
| B14598 | TACTCCATCACCACCCACAA | *STE20* Northern Primer NP2 |
| B14599 | GGCCTCCCAAGGAGATTTAG | *STE11* Northern Primer NP1 |
| B14600 | CTCGAAAGGAGGAAGTGACG | *STE11* Northern Primer NP2 |
| B14601 | TATGGGTCTGAGGACGCTTT | *STE50* Northern Primer NP1 |
| B14602 | GGTGTTTGCCCATCTTCTGT | *STE50* Northern Primer NP2 |
| 6055 | AGGGACAGTGACCAAAGTG | *STE7* Northern Primer NP1 |
| 6056 | TCTGACTGACACCGTTACG | *STE7* Northern Primer NP2 |
| B14603 | ACCTCAAGCCCTCAAACCTT | *CPK1* Northern Primer NP1 |
| B14604 | TCAGCAGTAGGTTCGTCGTG | *CPK1* Northern Primer NP2 |
| B14605 | GAAGGCTCGGGGAATAAGTC | *MAT2* Northern Primer NP1 |
| B14606 | GTAGCTGGCAAAGGAGCATC | *MAT2* Northern Primer NP2 |
| B14607 | GACCGAAACAAGCACGGTAT | *ZNF2* Northern Primre NP1 |
| B14608 | GATATTTGGGAGGCGTAGCA | *ZNF2* Northern Primre NP2 |
| B1894 | TTTTACGCTTTTTGCAGATTCCGCCAAA | *MFα1* Northern Primer NP1 |
| B1895 | GACCACTGTTTCTTTCGTTCT | *MFα1* Northern Primer NP2 |
| B13320 | ATGGAAGAAGAAGGTACG | *ACT1* Northern Primer NP1 |
| B13321 | TTAGAAACACTTTCGGTG | *ACT1* Northern Primer NP2 |
| B12350 | CGCGCCGCATGCTGCGGCCGCCGCAGAGGCTTGGGC | *HAP2* 4xFLAG-tagged strain Fwd |
| B12351 | GATGCATGCTCGAGCGGCCGCGGGTAAGAGCGGCATTG | *HAP2* 4xFLAG-tagged strain Rev |
| B12619 | TAGTGGCCGGCATTCAATTC | *GPA2* ChIP-PCR qRT Primer qRT1 |
| B12620 | GTGAATTACTCGCTACTCACGG | *GPA2* ChIP-PCR qRT Primer qRT2 |
| B12621 | TGAGTTACAAATCACAGGCCT | *CRG1* ChIP-PCR qRT Primer qRT1 |
| B12622 | CCAGATCAACTCACATCGGC | *CRG1* ChIP-PCR qRT Primer qRT2 |
| B12623 | TGAGTGAAAGTGGCTCATCG | *TUB1* ChIP-PCR qRT Primer qRT1 |
| B12624 | AGCAAGCCAAAAACAACACC | *TUB1* ChIP-PCR qRT Primer qRT2 |
