# Supplementary material for: Role of the Heme Activator Protein Complex in the Sexual Development of Cryptococcus neoformans
Source: mSphere. 2022 May 31;7(3):e00170-22. doi: 10.1128/msphere.00170-22 (PMC9241503; doi:10.1128/msphere.00170-22)
Supplement: FIG S8 [file msphere.00170-22-sf008.pdf]

Fig S8

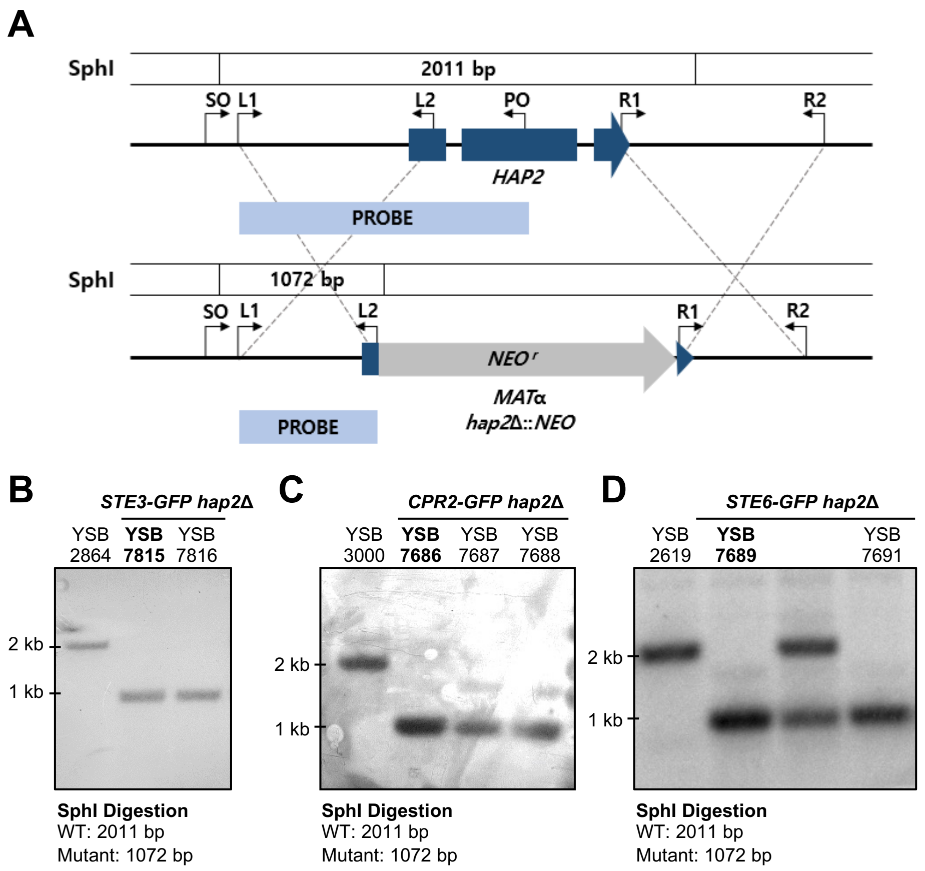

Fig S8. Construction of *STE3-GFP hap2*Δ, *CPR2-GFP hap2*Δ, and *STE6-GFP hap2*Δ mutant strains

(A) Diagram of the *HAP2* gene disruption strategy in the *MAT*α *STE3-GFP, MAT*α *CPR2-GFP*, and *MAT*α *STE6-GFP* strain. (B-D) Confirmation of the *STE3-GFP hap2*Δ, *CPR2-GFP hap2*Δ, and *STE6-GFP hap2*Δ gene disruption using Southern blot analysis.
